# An LES Turbulent Inflow Generator using A Recycling and Rescaling Method

**DOI:** 10.1007/s10494-016-9778-6

**Published:** 2016-10-31

**Authors:** F. Xiao, M. Dianat, J. J. McGuirk

**Affiliations:** 1 Science and Technology on Scramjet Laboratory, College of Aerospace Science and Engineering, National University of Defense Technology, Changsha, 410073 China; 2 Department of Aeronautical and Automotive Engineering, Loughborough University, Loughborough, LE11 3TU UK

**Keywords:** Turbulent inflow generator, Recycling, Rescaling, Turbulent mixing layer, Droplet dispersion

## Abstract

The present paper describes a recycling and rescaling method for generating turbulent inflow conditions for Large Eddy Simulation. The method is first validated by simulating a turbulent boundary layer and a turbulent mixing layer. It is demonstrated that, with input specification of mean velocities and turbulence rms levels (normal stresses) only, it can produce realistic and self-consistent turbulence structures. Comparison of shear stress and integral length scale indicates the success of the method in generating turbulent 1-point and 2-point correlations not specified in the input data. With the turbulent inlet conditions generated by this method, the growth rate of the turbulent boundary/mixing layer is properly predicted. Furthermore, the method can be used for the more complex inlet boundary flow types commonly found in industrial applications, which is demonstrated by generating non-equilibrium turbulent inflow and spanwise inhomogeneous inflow. As a final illustration of the benefits brought by this approach, a droplet-laden mixing layer is simulated. The dispersion of droplets in the near-field immediately downstream of the splitter plate trailing edge where the turbulent mixing layer begins is accurately reproduced due to the realistic turbulent structures captured by the recycling/rescaling method.

## Introduction

Many engineering applications require accurate prediction of turbulent mixing rates. One important example is the mixing and combustion of fuel and air in gas-turbine or IC engines. Fuel and air are often introduced separately and rapidly mixed and burnt in combustion zones. Whether the fuel is in gaseous or liquid (droplet) form it is important to capture the rate of turbulent mixing right from the point where fuel and air first come into contact, particularly when ignition and flame stabilisation performance are of interest. Whilst Large Eddy Simulation (LES) is now widely regarded as a better approach for prediction of turbulent mixing in complex flows than conventional RANS turbulence modelling, this requirement for high accuracy right from the origin of the mixing region simulation is a particularly challenging task for LES, where the optimum approach to inlet boundary condition treatment is still under development.

It has been realised for some time that the quality of specification of LES inlet conditions can exert a significant influence on the simulation accuracy, especially in the region close to the inlet boundary, although this influence can also persist over large downstream distances. Tyacke and Tucker [33], for example, have emphasised the importance of a generally applicable inflow generation method for the complex flows found in turbomachines. Many other authors have observed how inlet conditions affect the predicted flow development, for example Lund et al. [19] for boundary layers, Xiao et al. [38–40] for liquid jet primary breakup, and McMullan et al. [22] for free shear layers. Reviews of the various approaches suggested for specification/generation of LES inlet conditions can be found in Baba-Ahmadi and Tabor [4], Tabor and Baba-Ahmadi [30] and most recently by Wu [37]; in all of these studies great emphasis has been placed on the ability of the proposed method to shorten the adjustment length i.e. the distance downstream of the inlet boundary where the specified inlet unsteady fluctuations adjust to become completely consistently correlated.

In general, two approaches have been followed: (i) synthetic methods, where spatially and temporally correlated unsteady fluctuations, generated in a variety of ways, are superimposed on an inlet mean velocity field, and (ii) recycling/rescaling methods, where a separate (auxiliary) LES simulation is performed, the unsteady velocity field extracted at some selected plane in the auxiliary domain, rescaled, and then imposed at the inlet boundary of the auxiliary domain; the inlet boundary condition for the actual (main) simulation domain is transferred from a selected location within the auxiliary domain to the main simulation inlet boundary. For correct reproduction of the flow development in the immediate near-field of the main simulation inlet boundary, it is necessary to specify realistic time-series at the LES inlet boundary (i.e. correctly correlated and an accurate representation of the local flow conditions for both time-mean velocity and turbulent fluctuations).

Among the synthetic methods, the most straightforward is to superimpose white noise on an assumed or known mean velocity field (e.g. from experimental measurements or a RANS simulation). However, the uncorrelated nature of white noise means the generated turbulence lacks large-scale energy containing structures. Such pseudo turbulence is dissipated rapidly downstream of the inflow plane, and a adjustment region of considerable length is then needed to recover realistic and self-consistent turbulence structures. Several authors have suggested methods whereby some turbulence information is provided at the inlet plane and used to generate a more detailed and physically realistic data set of unsteady inlet velocity conditions. Lee et al. [17] used an inverse Fourier transform of an assumed energy spectrum to reconstruct turbulent fluctuations, but the lack of phase information of real eddies proved problematic. Batten [5] suggested generating turbulent fluctuations from a summation of sine and cosine functions with random phases and amplitudes. Keating et al. [13] applied this method in a turbulent plane-channel flow, but still observed very slow development of turbulence structures until they also adopted the controlled forcing method of Spille-Kohoff [29]. Jarrin et al. [10] generated synthetic turbulence by directly prescribing coherent modes; this produced the correct friction factor in a channel flow, but still required a length of 6 channel heights to achieve this. Kempf et al. [14] proposed a method that converts white noise into a signal featuring the required length-scale through a diffusion process. Finally, what has developed into perhaps the most popular approach of this type is the technique based on generation of a digital filter whose coefficients are adjusted to fit specified 1st and 2nd moment one-point statistics, together with an assumed length scale and a Gaussian 2-point correlation function - Klein et al. [15], di Mare et al. [20], Veloudis et al. [35].

All of the above synthetic turbulence generation approaches have two drawbacks. Firstly, the adjustment region problem mentioned above is always present to some extent. Lund et al. [19] found that a development region of some 50 boundary layer thicknesses was required for a wall boundary layer; Le et al. [16] observed that their method required about 10 step heights for attainment of physically consistent turbulence characteristics in a backward-facing step flow. Secondly, most of the more advanced methods demand an input of turbulence information that is only rarely available, for example turbulence length scales or correlation shapes.

These problems are avoided by methods based on the recycling technique. This approach was first adopted for fully-developed duct flows, where periodic boundary conditions (a form of spatial recycling) between inlet and outlet may be used. By applying a carefully designed coordinate transform, Spalart [27] was able to use the recycling approach for DNS of a spatially developing boundary layer. In the transformed frame the velocity field is approximately homogeneous in the main flow direction so periodic conditions can be applied. However, during the coordinate transformation, some extra complex terms are added to the Navier-Stokes equations to account for the inhomogeneity in the streamwise direction. Furthermore, the streamwise gradients of the mean velocity included in these terms need to be specified explicitly. Therefore, Spalart’s method is complex to program.

Lund et al. [19] produced a simplified version of Spalart’s method requiring no coordinate frame transformation. During the LES solution, an instantaneous velocity field was extracted from a plane near the solution domain exit, rescaled according to self-similarity laws for boundary layers (e.g. mean flow scaled following the law of the wall/defect law in the inner/outer regions respectively, fluctuations rescaled using local friction velocities) and then recycled upstream to form the inflow conditions at solution domain inlet. This method is substantially simpler, but is, however, still only applicable to boundary layer-type flows because of the particular rescaling concepts adopted. Lund et al. [19] implemented their method in a separate (precursor) LES calculation and imported the eventual inlet conditions for a main calculation from this. Mayor et al. [21] applied the same method, but realised it could be implemented by merging the inflow generation procedure with the main simulation. However, some aspects of Lund et al.’s method can make it difficult to implement [11]. Improved variants of Lund et al.’s approach have continued to be developed, modifying problems identified in both the rescaling and recycling elements, although to date still constructed primarily with spatially developing boundary layers in mind. For example, Liu and Pletcher [18] addressed the problem that the rescaling operation is based on the similarity laws of the boundary layer and, if the downstream data at the recycling plane have not yet reached their equilibrium state, the similarity laws do not strictly apply and incorrect data are recycled, leading to a longer adjustment length and start-up transient. A dynamic procedure was proposed where the recycling plane was initially placed close to the inlet plane, and only moved downstream gradually. An alternative dynamic approach has been suggested by Araya et al. [2] using 3 planes (inlet, test and recycling) rather than two and adopting modified scaling laws. Both of these ideas shorten the adjustment length. A second problem with the recycling/rescaling procedure is that a recycling process between two spatially separate planes will inevitably introduce a non-physical spatial/temporal correlation into the data generated, as demonstrated by Nikitin [24]. Spalart et al. [28], Jewkes et al. [11], and Morgan et al. [23] have investigated methods to remove (or at least reduce) this, which involve introduction of techniques for spanwise scrambling of the data before recycling. This approach has also been successfully implemented in an unstructured CFD code by Arolla [3] and applied to turbomachinery flows. These methods are successful but unfortunately currently limited to flows with spanwise homogeneity. This numerical artefact of recycling/rescaling techniques will not be too problematic if the unphysical frequency introduced has only small energy relative to the true turbulent frequencies of the larger eddies most responsible for mixing, however this is an unwanted feature of recycling/rescaling algorithms and needs to be carefully examined when these are applied until a general method to eliminate this problem is identified.

All the above implementations of the recycling/rescaling technique have inherently been restricted to boundary layers. Pierce [25] proposed a quite different rescaling approach which generalised the recycling technique for any inflow profile. Rather than rescale the velocity field of one specified plane to act as the inflow velocities at the inlet plane as in Lund et al. [19], Pierce [25] rescaled the velocity of the whole inflow generation region to constrain the velocity so that the generated velocity field within the inflow simulation domain has user-defined velocity statistics profiles (in particular 1*s* *t* moment (mean) velocity and 2*n* *d* moment normal stress). With this recycling/rescaling technique, all spatial and temporal correlations characterising the turbulence structures are self-generated and self-consistent with the pre-specified 1st and 2nd moment statistics. Due to the advantages of this technique, a recycling and rescaling method (hereafter referred to as R^2^M) based on the work of Pierce [25], Lund [19] and Spalart [27] is further studied and tested in the current work. The following section describes the LES methodology and code adopted and the algorithm of the R^2^M approach proposed in the current work in detail. Since mixing regions in practical applications are often associated with a mixing layer formed from the merger of upstream boundary layers, the method is first validated by simulating a spanwise homogeneous turbulent boundary layer and then a mixing layer growing from two merging boundary layers. It is then shown how the method can be used for inlet profiles which depend on both spanwise and transverse co-ordinates. Finally, as an illustration of the benefits that can be achieved using the present method, it is applied to the problem of dispersion of liquid droplets across a turbulent mixing layer. The measurements of Tageldin and Cetegen [31] are used to illustrate the improvements using the present R^2^M approach in the near-field of the splitter plate from which the mixing layer grows. This problem has previously been studied using LES by Jones et al. [12] and the present methodology is therefore contrasted with this prior work to assess its performance.

## Methodology

### LES algorithm

The LES code used in the present work is an incompressible, pressure-based method. The code (LULES) is based on solving a transformed version of the Cartesian transport equations using a curvilinear orthogonal co-ordinate system and contravariant velocity decomposition. The spatially filtered transport equations are discretised using the finite volume method. For the momentum equations, spatial derivatives for both convective and diffusive terms are calculated using a second order central differencing scheme; the Adams-Bashforth second-order explicit scheme is used for temporal discretisation. The LES code uses a structured, multi-block, staggered mesh and a multi-grid method to speed up solution of the Poisson equation solved to satisfy continuity. A standard Smagorinsky SGS model is incorporated, using Smagorinsky coefficient *C*_*S*_=0.1, a length scale based on the cube root of the cell volume, and a Van Driest near wall damping function. Details of the transformed equations, discretisation practices, and basic testing/validation of the code have been described fully in Tang et al. [32] and Dianat et al. [7]. The inlet condition methodology is here described as implemented in such a multi-block, structured mesh code, but extension to other mesh types is clearly possible.

### Droplet Lagrangian tracking method

The code described above is appropriate when the flow problem comprises a single phase, e.g. gaseous flow. For the final flow problem reported below, a two-phase flow is considered, where the dispersion of discrete liquid droplets in a turbulent air flow is analysed and compared against experimental data. In the present work, the motion of droplets is tracked using Lagrangian approach for the liquid phase and an Eulerian approach for the gas phase. The droplets are assumed to remain spherical, and the droplet velocity and location are governed by [6]:

1 $$ \frac{\mathrm{d}\boldsymbol{u}_{\boldsymbol{d}}}{\mathrm{d}t}=\frac{f}{\tau_{V}}\boldsymbol{u}_{\boldsymbol{r}}+\boldsymbol{g} \\ $$

2 $$ \frac{\mathrm{d}\boldsymbol{x}_{\boldsymbol{d}}}{\mathrm{d}t}=\boldsymbol{u}_{\boldsymbol{d}} $$

where ***u***_***d***_ and ***x***_***d***_ are droplet velocity and location respectively, ***u***_***r***_ the gas velocity relative to the droplet (***u***_***r***_ = ***U***−***u***_***d***_ where ***U*** is the gas velocity at the droplet position), ***g*** is the gravitational acceleration. *τ*_*V*_ is the velocity response time which is defined by *τ*_*V*_ = *ρ*_*d*_ *D* ^2^/(18*μ*_*g*_). *D* is droplet diameter, *ρ*_*d*_ is the droplet density, and *μ*_*g*_ is the dynamic viscosity of the gas. *f* is the drag factor which is the ratio of the drag coefficient to Stokes drag *f* = *C*_*D*_Re_*r*_/24. *C*_*D*_ is drag coefficient, and Re_*r*_ is the droplet Reynolds number defined by $\text {Re}_{r}=\rho _{g}\left |\boldsymbol {u}_{\boldsymbol {r}}\right |D/\mu $. *ρ*_*g*_ is the gas density. Since the droplet Reynolds number is of the order of 10 in the simulated test case, the correlation for *f* proposed by Schiller and Naumann [26] which is reasonably good for Reynolds numbers up to, 800 is used:

3 $$ f=1+0.15\text{Re}_{r}^{0.687} $$

In LES predictions the resolved unsteady motion of the large scale turbulent eddies is primarily responsible for turbulent dispersion of the droplets. Depending on the grid size chosen this may be sufficient to capture the dispersion process adequately. However, if significant turbulent energy is present in the unresolved (sub-grid) scales, then it may be necessary also to include a model for subgrid-scale (SGS) droplet dispersion. Following the work described in Jones et al. [12], the influence of the unresolved gas phase fluctuations has been modelled by a stochastic Wiener process, which is added to the deterministic contribution:

4 $$ \mathrm{d}\boldsymbol{{u}_{d}}=\frac{f}{\tau_{V}}\boldsymbol{{u}_{r}}{\mathrm{d}t}+\boldsymbol{g}{\mathrm{d}t}+\left( C_{0}\frac{k_{SGS}}{\tau_{t}}\right)^{1/2}\mathrm{d}\boldsymbol{{W}_{t}} \quad \tau_{t}=\tau_{V} \left( \frac{\tau_{V} \sqrt{k_{SGS}}}{\Delta}\right)^{2\alpha-1} $$

where ***W***_***t***_ is the increment of the Wiener process, *k*_*SGS*_ is an estimate for the SGS turbulent kinetic energy, and Δ is the LES filter width (taken here to be the cube root of the local cell volume). An equilibrium estimate is used for $k_{SGS}=\mu _{SGS}\left (2\bar {S}_{ij}\bar {S}_{ij}\right )^{1/2}$, where $\bar {S}_{ij}$ is the strain rate calculated using the resolved gas velocity $\frac {1}{2}\left (\frac {\partial U_{i}}{\partial x_{j}}+\frac {\partial U_{j}}{\partial x_{i}}\right )$. Jones et al. [12] have studied the effect of varying the model parameters *C*_0_ and *α* on the droplet dispersion, and *C*_0_=1.0 and *α*=0.5 are chosen here basing on their recommendation.

### Recycling and rescaling method (R ^2^M)

**Fig. 1:**
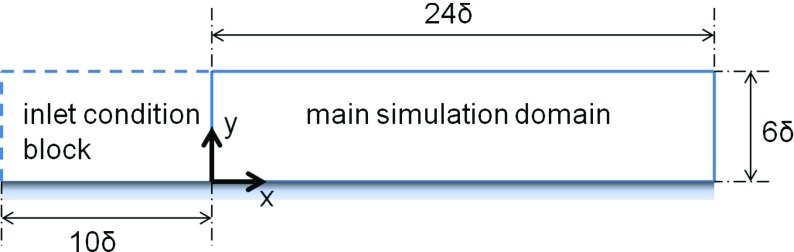
Structure of the simulation domains for a turbulent boundary layer

The proposed inflow generation method for some of the LES solutions presented below makes use of a recycling and rescaling technique. First, an extra “inlet condition” (IC) domain (single block or multi-blocks as necessary) is created upstream of the real inlet-plane of the main simulation (MS) domain, see Fig. 1 for a turbulent boundary layer. The inflow conditions for the extra IC domain are generated by recycling the velocity field from a selected plane in the downstream region of the IC domain. In order to generate realistic unsteady inflow for LES using R^2^M, target values for the mean velocity and Reynolds normal stress (rms intensity) profiles at the MS domain inlet-plane need to be prescribed, i.e., $\bar {U}_{target}(y),\,$ $ \bar {V}_{target}(y),\, $ $\bar {W}_{target}(y)\,$, $ u_{target}^{\prime }(y),\, $ $v_{target}^{\prime }(y) ,\,$ $ w_{target}^{\prime }(y) $ for spanwise homogeneous inflow conditions or $\bar {U}_{target}(y,z),\,$ $ \bar {V}_{target}(y,z),\,$ $ \bar {W}_{target}(y,z),\, $ $u_{target}^{\prime }(y,z),\,$ $ v_{target}^{\prime }(y,z) ,\, $ $w_{target}^{\prime }(y,z)$ for spanwise inhomogeneous inflow conditions. The target values can be from either experiments or numerical simulations (usually RANS, but possibly even LES or DNS). By rescaling the velocities, the resulting instantaneous flow field within the IC domain can achieve the target statistical characteristics whilst also possessing self-consistent spatial and temporal correlations.

**Fig. 2:**
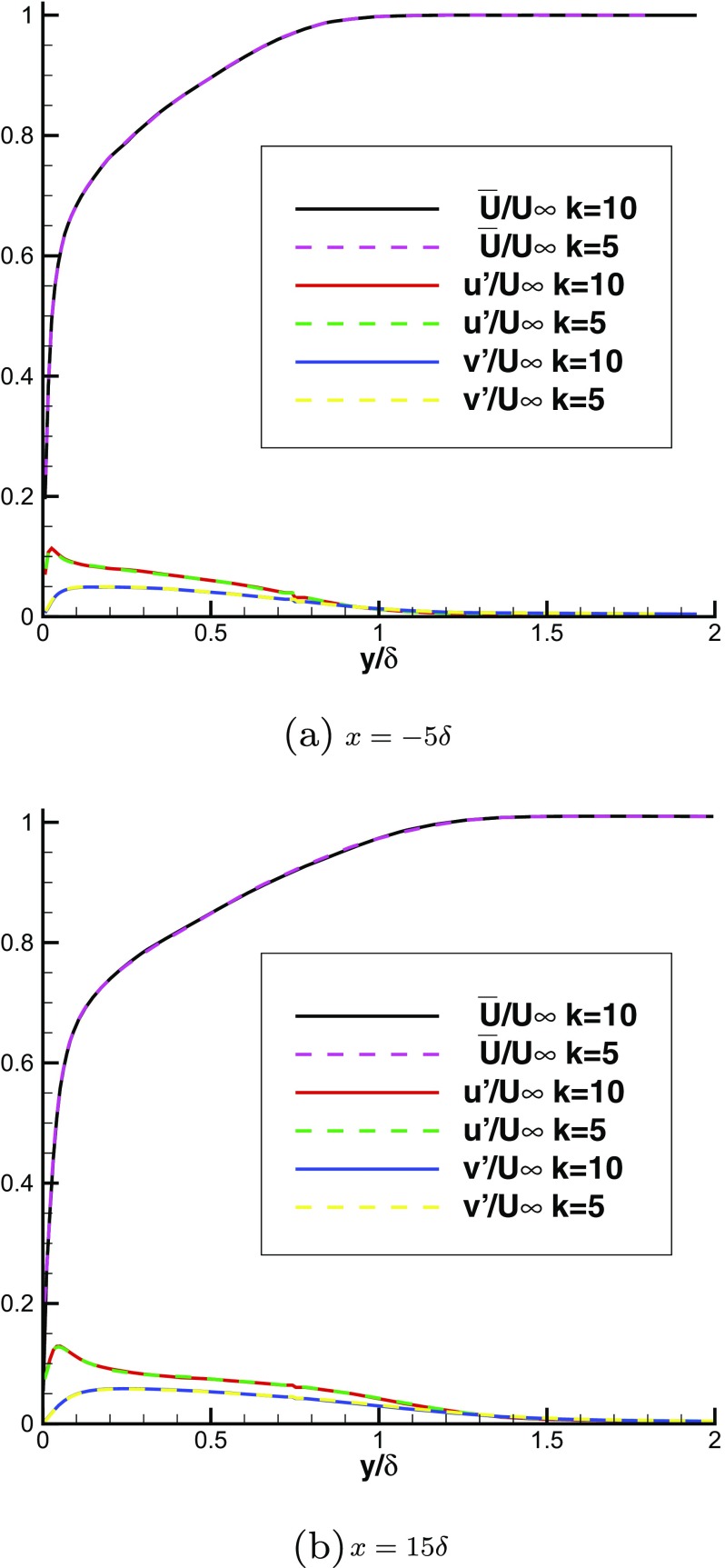
Comparison of the mean velocity and rms intensity predicted with k=5 and k=10

The procedure for generating LES inflow conditions is first described for the case of spanwise (i.e. *z* direction) homogeneous conditions:

1. Create an extra IC domain (1 or more blocks) upstream of the MS inlet-plane where turbulent inflow needs to be specified. The size of the IC domain in the spanwise (*z*) and transverse (*y*) directions is usually fixed by the MS inlet-plane size for convenience. (When the IC domain has a different size in the *z* and *y* directions from the MS inlet-plane, a mapping procedure is needed as demonstrated in [40].) The size in the streamwise (*x*) direction is chosen so that the two point spatial correlations fall to zero well within the IC block, as required by the recycling technique.
2. Use the recycling method to provide inflow conditions for the IC block. The velocity field at a plane a short distance upstream of the real MS domain inlet-plane is recycled. This is to avoid any upstream influence of the flow development in the MS domain. In addition, it is important that the mesh in the IC domain should be uniform in the *x* and *z* directions (homogeneous directions) to avoid any spatially-varying scaling effects.
3. Initialise the velocity field in the IC domain as well as in the MS domain. When initialising in the IC domain, the instantaneous velocity field is generated by superimposing white noise with an intensity of $ u_{target}^{\prime }(y)$, $v_{target}^{\prime }(y)$, $ w_{target}^{\prime }(y) $ on the mean velocity $\bar {U}_{target}(y),\, \bar {V}_{target}(y),\, \bar {W}_{target}(y)$.
4. Run the simulation in both IC and MS domains simultaneously. Rescale the flow field everywhere within the IC domain every k LES time steps as shown below. blackNote: it has been mentioned in the Introduction that the frequency of the recycling/rescaling operation can introduce spurious frequencies into the simulation. The amplitude of these is of concern and will be investigated below, but to demonstrate that this choice has no effect on statistical quantities, two simulations with k=5 and k=10 have been carried out. Figure 2 shows that these produce the same mean velocity and turbulence level, following this test k =10 was used in all other simulations presented.
  Calculate the mean velocity by spatial averaging in the *x* and *z* directions and temporal averaging with a weight that decreases exponentially backward in time (see [19] for more about temporal averaging):
    5 $$\begin{array}{@{}rcl@{}} \bar{U}^{(n+1)}(y)&=&\frac{k\triangle t}{T}\langle U(x,y,z,t)\rangle_{x-z}+\left( 1-\frac{k\triangle t}{T}\right)\bar{U}^{n}(y) \end{array} $$
    6 $$\begin{array}{@{}rcl@{}} &=&\frac{k\triangle t}{T}\frac{1}{PQ}{\sum}_{i=1}^{P}{\sum}_{j=1}^{Q}U(x_{i},y,z_{j},t)+\left( 1-\frac{k\triangle t}{T}\right)\bar{U}^{n}(y) \end{array} $$
    Where △*t* is the computational time step, *T* is a characteristic time scale for the temporal averaging which will be examined below, 〈 〉_*x*−*z*_ represents spatial averaging in the *x*−*z* plane, and *U*(*x*,*y*,*z*,*t*) is the current instantaneous solution.
  Calculate the rms of the velocity field in a similar way
    7 $$ u^{{\prime}(n+1)}(y)=\sqrt{\frac{k\triangle t}{T}\left\langle \left[U(x,y,z,t)-\bar{U}^{(n+1)}(y)\right]^{2}\right\rangle_{x-z}+\left( 1-\frac{k\triangle t}{T}\right) \left[u^{{\prime}n}(y)\right]^{2}} $$
  Rescale the instantaneous velocity to create a new instantaneous velocity field:
    8 $$\begin{array}{@{}rcl@{}} U^{new}(x_{i},y,z_{j},t)&=&\frac{u^{\prime}_{target}(y)}{u^{{\prime}(n+1)}(y)}[U(x_{i},y,z_{j},t)-\bar{U}^{(n+1)}(y)]+\bar{U}_{target}(y)\\ i&=&1,P;\quad j=1,Q \end{array} $$
  Rescale the other two velocity components *V* and *W* following the same procedure.

For spanwise inhomogeneous inflow conditions, the sequence of steps is similar, but modified as follows:

1. Create an extra IC domain and use the recycling method in the same way as above.
2. Initialise the velocity field in IC and MS domains. When initialising in the IC domain, the instantaneous velocity field is again generated by superimposing white noise with an intensity of $u_{target}^{\prime }(y,z)$, $v_{target}^{\prime }(y,z)$, $w_{target}^{\prime }(y,z) $ on the user-specified mean velocity $\bar {U}_{target}(y,z),\, \bar {V}_{target}(y,z),\, \bar {W}_{target}(y,z) $.
3. Run the simulation in both IC and MS domains simultaneously. Rescale the flow field in the IC domain every k time steps in the following way:
  Calculate the mean velocity by spatial averaging but now in the streamwise (*x*) direction only and temporal averaging:
    9 $$\begin{array}{@{}rcl@{}} \bar{U}^{(n+1)}(y,z)&=\frac{k\triangle t}{T}\langle U(x,y,z,t)\rangle_{x}+\left( 1-\frac{k\triangle t}{T}\right)\bar{U}^{n}(y,z) \end{array} $$
    Where 〈 〉_*x*_ represents spatial averaging in the streamwise direction.
  Calculate the rms of the velocity field in a similar way:
    10 $$ \begin{array}{ll} &u^{{\prime}(n+1)}(y,z)=\\ &\sqrt{\frac{k\triangle t}{T}\left\langle \left[U(x,y,z,t)-\bar{U}^{(n+1)}(y,z)\right]^{2}\right\rangle_{x}+ \left( 1-\frac{k\triangle t}{T}\right) \left[u^{{\prime}n}(y,z)\right]^{2}} \end{array} $$
  Rescale the instantaneous velocity to create a new instantaneous velocity field:
    11 $$ U^{new}(x_{i},y,z,t)=\frac{u^{\prime}_{target}(y,z)}{u^{{\prime}(n+1)}(y,z)}[U(x_{i},y,z,t)-\bar{U}^{(n+1)}(y,z)]+\bar{U}_{target}(y,z) $$
  Rescale the other velocity components *V* and *W* following the same procedure.

**Fig. 3:**
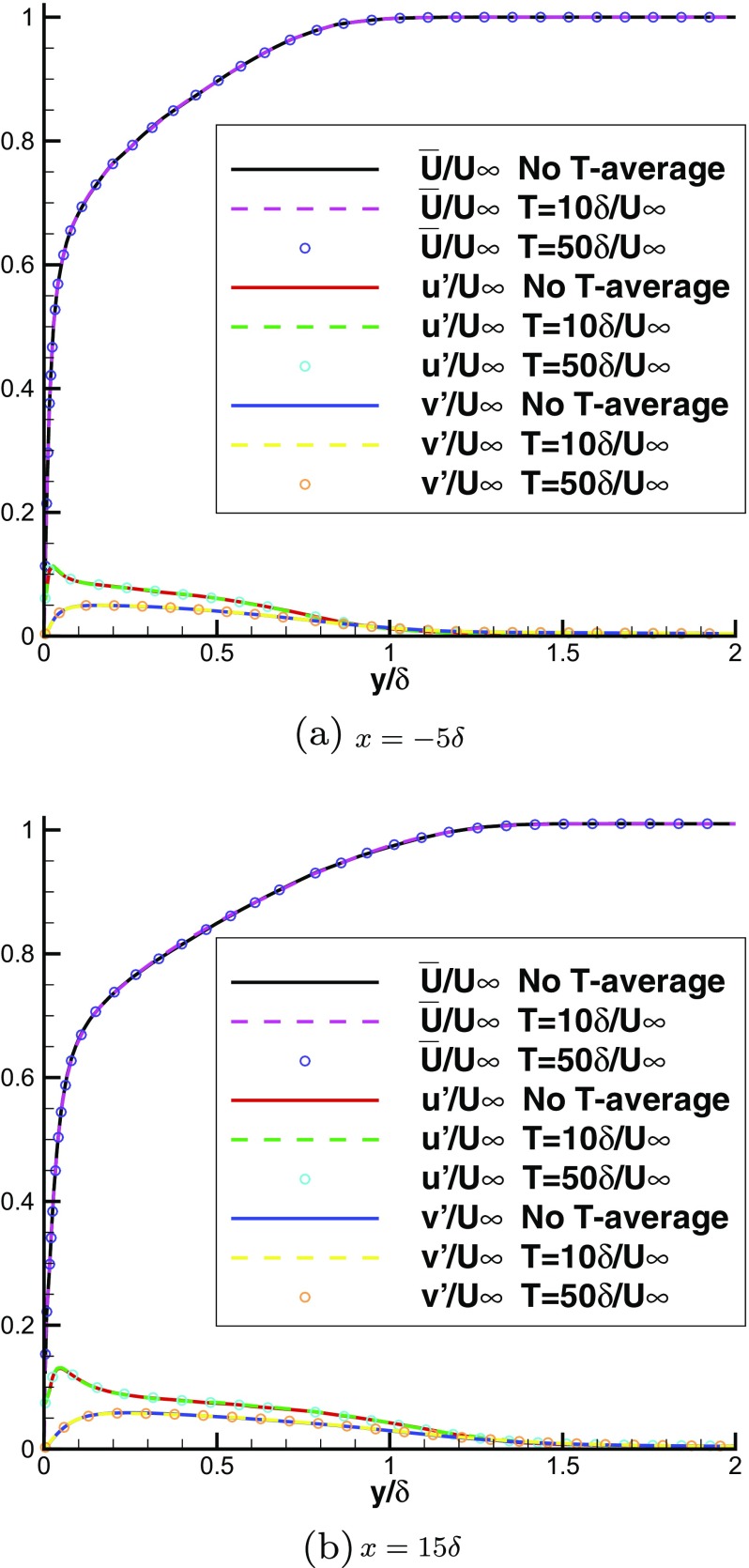
Comparison of the mean velocity and rms intensity predicted with temporal averaging ($T=10\delta /U_{\infty }$, $T=50\delta /U_{\infty }$) and without temporal averaging (No T-average)

**N.B.** When generating spanwise homogeneous inflow conditions, the velocity statistics could be calculated by only spatial averaging in the x-z plane without the temporal averaging. In this case, almost the same results were obtained as with both spatial and temporal averaging as shown in Fig. 3, indicating that spatial averaging in the streamwise and spanwise directions is sufficient to obtain the correct statistics of velocities. Figure 3 also demonstrates that the two simulations with $T=10\delta /U_{\infty }$ and $T=50\delta /U_{\infty }$ produce the same mean velocity and rms intensity in both IC and MS domains, indicating that the choice of T has little influence when generating spanwise homogeneous turbulent inflows by R^2^M. However, when generating spanwise inhomogeneous inflow conditions, spatial averaging can only be carried out in the streamwise direction, making temporal averaging indispensable to obtain converged velocity statistics efficiently; readers are referred to [19] for more details on temporal averaging, and $T=10\delta /U_{\infty }$ is used in the following simulations.

## Results

### LES of a turbulent boundary layer

**Fig. 4:**
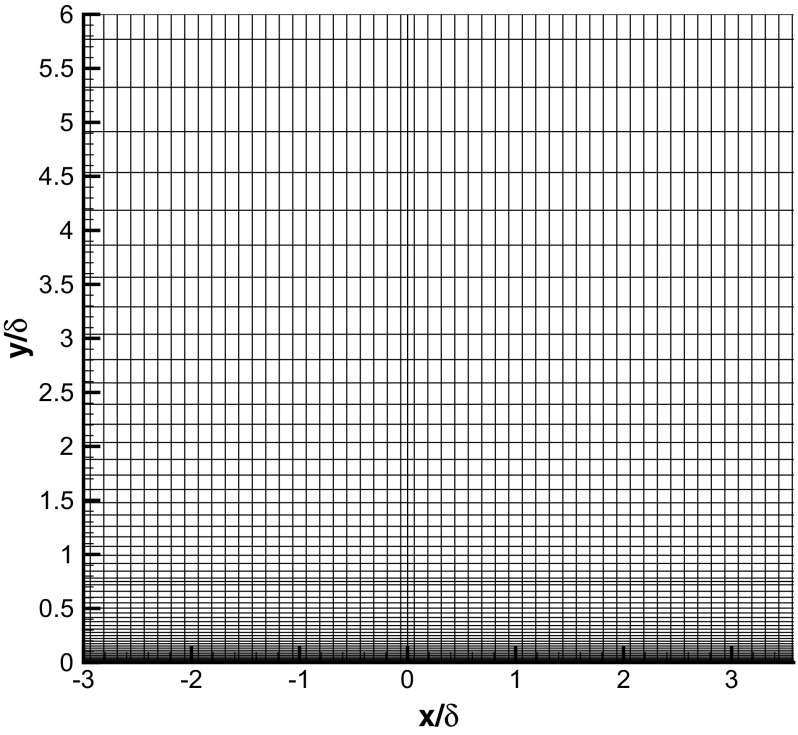
Mesh for a turbulent boundary layer

It is well known in LES/DNS that the development of flow in a turbulent boundary layer is very sensitive to the quality of the specified inflow. Therefore, a spanwise homogeneous turbulent boundary layer is an appropriate first test case to demonstrate the proposed recycling-rescaling method. Figure 1 shows the main simulation domain as well as the extra inlet condition domain. The main simulation domain is nearly the same as that used in Lund et al. [19] so that it is straightforward to compare the method proposed here with the methods surveyed in [19]. The only difference is that the size in the wall-normal direction is 6*δ* (*δ* is the 99 *%* boundary layer thickness at the inlet plane *x*=0) rather than 3*δ* as in [19]. The dimensions of the main simulation domain are 24*δ*×6*δ*×(*π*/2)*δ* in the streamwise, wall-normal and spanwise directions respectively , and the mesh contains 192×56×56 grid nodes. The dimensions of the IC domain are 10*δ*×6*δ*×(*π*/2)*δ* with the mesh containing 80×56×56 nodes. An example of the mesh in the upstream/downstream region of the MS inlet plane (*x*=0) is shown in Fig. 4. The mesh size expands in the transverse (*y*) direction with the near-wall minimum size Δ*y*_*min*_ equal to 0.01*δ*; the non-dimensional distance (*y* ^+^) of the first point from the wall is 2. The mesh is uniform in the streamwise (*x*) and spanwise (*z*)directions. The velocity field at the plane *x*=−2*δ* is recycled as the inflow conditions for the IC domain. The mean velocity and rms profiles of a boundary layer with *R* *e*_*𝜃*_=1410 from the DNS of Spalart [27] are used as the target values. Periodic boundary conditions are used in the spanwise (*z*) direction; a convective outflow boundary condition is applied at the outlet (*x*=24*δ*). And a zero-gradient boundary condition is used for the top surface of the simulation domain.

**Fig. 5:**
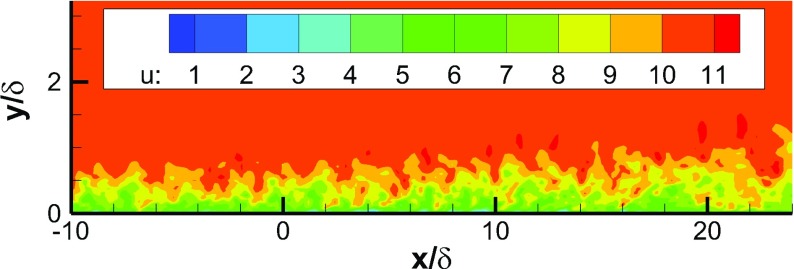
Contours of the streamwise velocity *U* in an *x*−*y* plane of a turbulent boundary layer simulation with recycling and rescaling method as inflow conditions for the region downstream of *x*/*δ*=0

Figure 5 shows the predicted instantaneous contours of the streamwise velocity *U* in an *x*−*y* plane. Coherent turbulent structures are developed in the IC domain. Due to the rescaling procedure, the boundary layer doesn’t grow within the IC domain. However, with the turbulent flow from the IC domain as the MS domain inflow condition, the boundary layer develops naturally in the MS domain, demonstrating the capability of the proposed method.

**Fig. 6:**
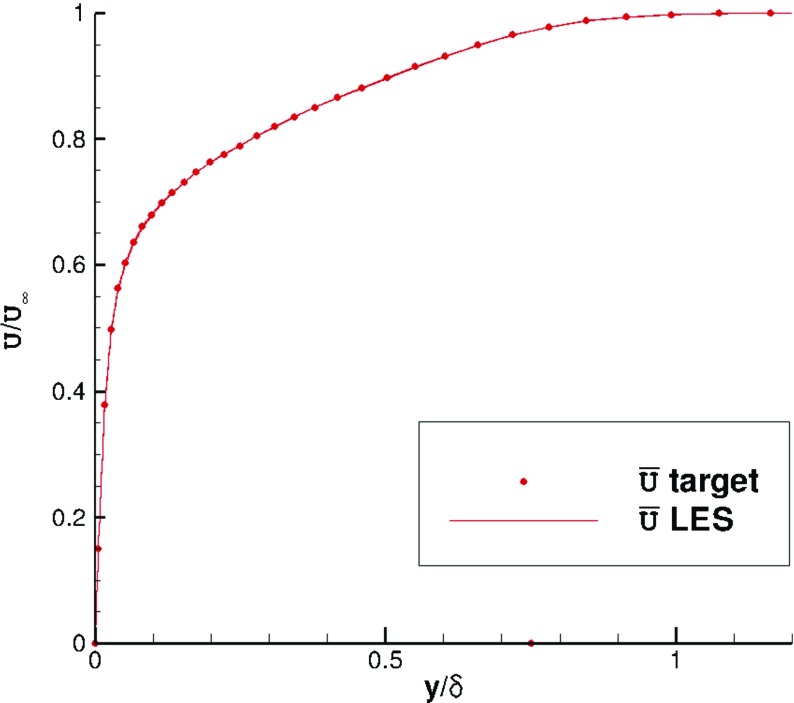
Mean velocity profiles at *x*=−*δ*,−5*δ*,−9*δ* in the IC domain for a turbulent boundary layer

**Fig. 7:**
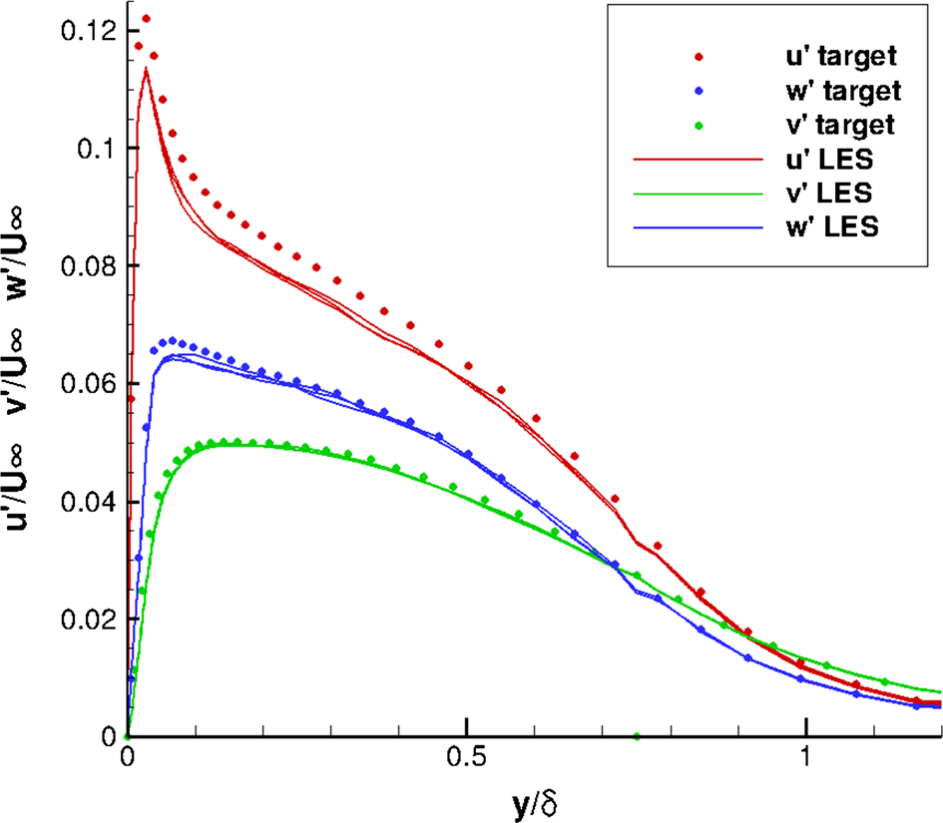
Rms profiles at *x*=−*δ*,−5*δ*,−9*δ* in the IC domain for a turbulent boundary layer

**Fig. 8:**
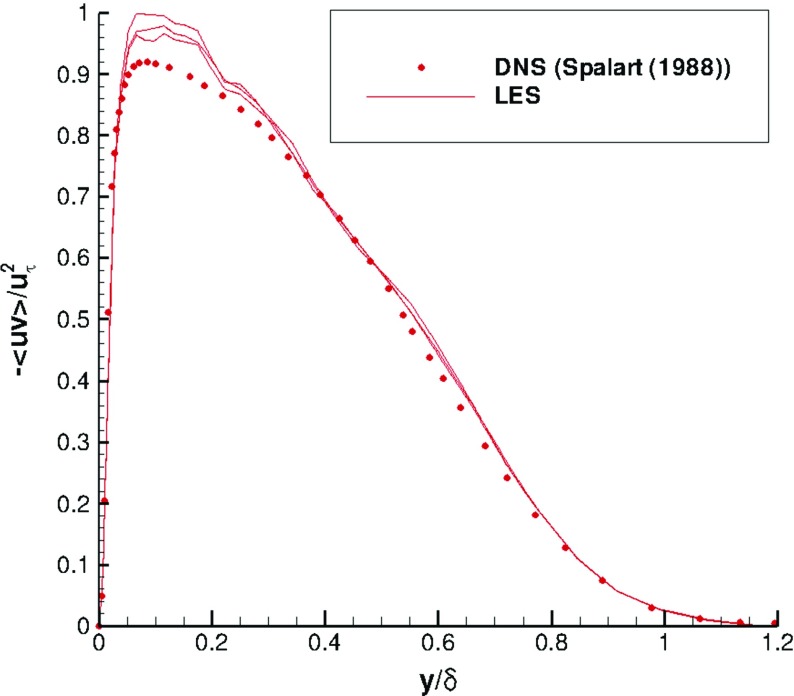
Shear stress profiles at *x*=−*δ*,−5*δ*,−9*δ* in the IC domain for a turbulent boundary layer

Figures 6 and 7 indicate that statistical mean velocity and rms levels in the IC domain fit the target values very well. The turbulent shear stress distribution, self-generated by R^2^M and not included in the input statistics, agrees well with the DNS of Spalart [27], as shown in Fig. 8. The statistical streamwise homogeneity within the IC domain solution is demonstrated in Figs. 6, 7 and 8, which include profiles for *x*=−*δ*, *x*=−5*δ* and *x*=−9*δ*. The near wall peak in shear stress is slightly overpredicted compared to the DNS data of Spalart [27], as might be expected from the simplicity of the SGS model used.

**Fig. 9:**
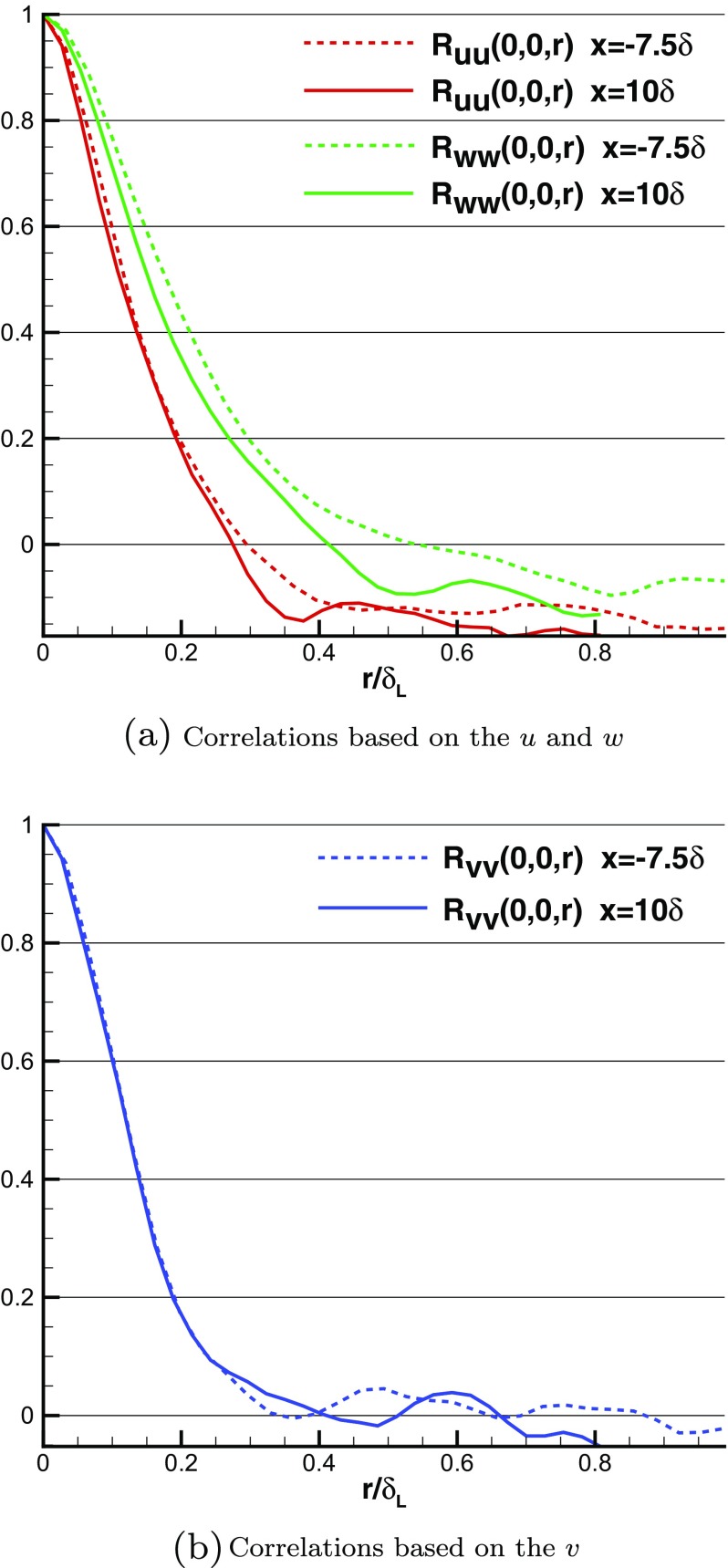
Comparison of 2-point spanwise spatial correlations at *y*=0.5*δ*_*L*_ in the IC and MS domains

**Fig. 10:**
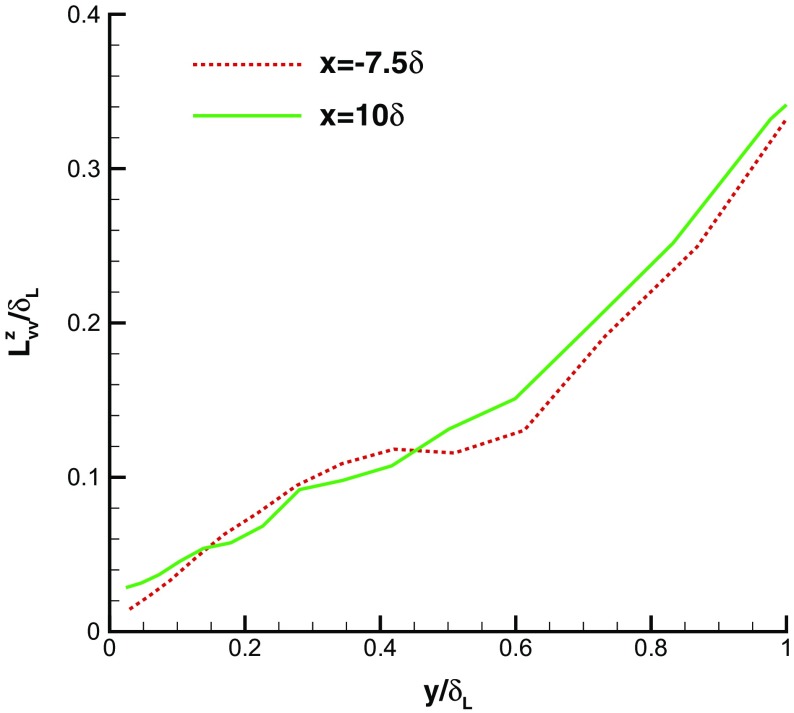
Spanwise integral lengthscale evaluated by integrating the spanwise spatial correlation for the *v*-component

**Fig. 11:**
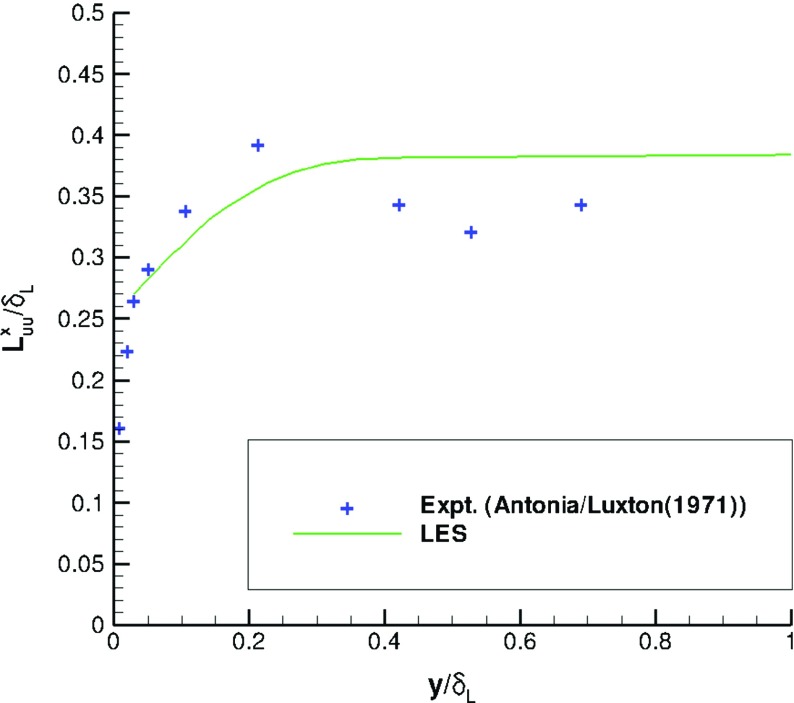
Streamwise integral length evaluated by integrating the *x*-direction spatial correlation for the *u*-component

**Fig. 12:**
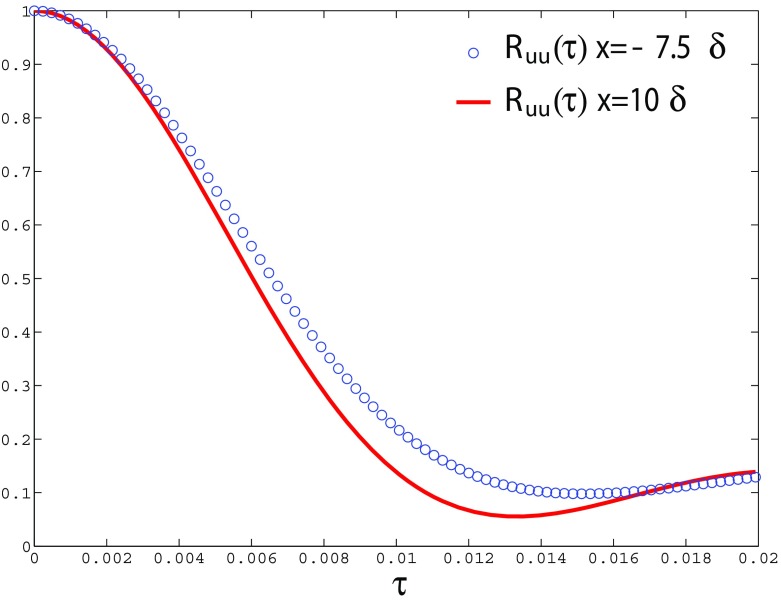
Comparison of the temporal correlation functions in the IC and MS domains (at y= 0.15*δ*)

**Fig. 13:**
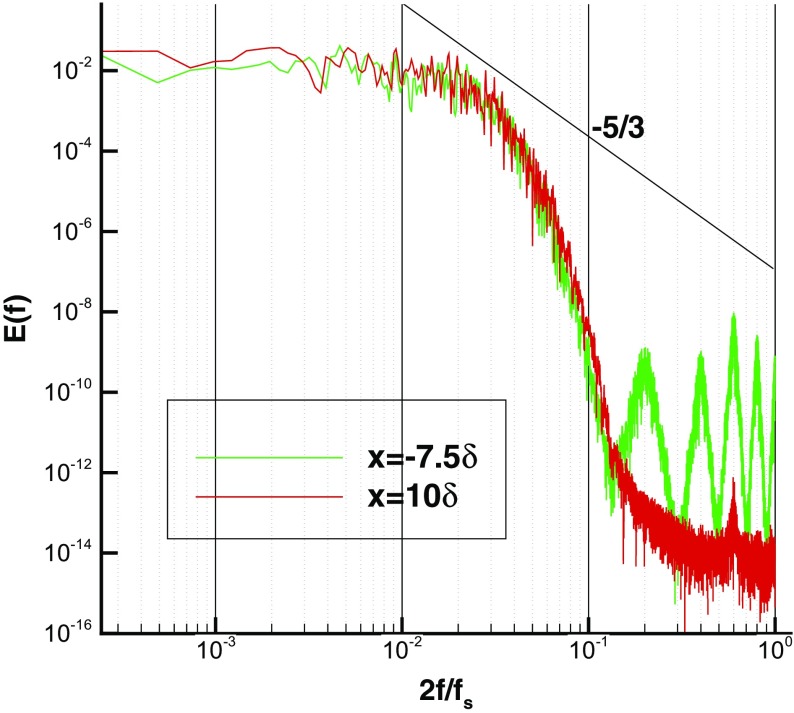
Comparison of the frequency spectra of the turbulent energy in the IC and MS domains (at y= 0.15*δ*)

Figure 9 compares spanwise 2-point spatial correlation functions at *y*=0.5*δ*_*L*_ (*δ*_*L*_ is the local 99 *%* boundary layer thickness). The correlation functions in the IC domain (*x*=−7.5*δ*) agree well with those in the MS domain (*x*=10*δ*), indicating that spatial coherence of turbulent flows is reproduced in the IC domain. Figure 10 shows the spanwise integral lengthscale evaluated by integrating the spanwise spatial correlation for the *v*-component. Good agreement is again observed for this integral lengthscale (scaled by *δ*_*L*_) between IC and MS domains, showing that the turbulent structures generated by R^2^M in the IC domain have the same nondimensional spatial lengthscale as turbulent flows governed by the N-S equations in the MS domain. Furthermore, the predicted streamwise integral length (evaluated by integrating the *x*-direction spatial correlation for the *u*-component) agrees well with experimental data measured via a hot-wire technique by Antonia and Luxton [1], as demonstrated in Fig. 11. Figure 12 shows the similarity of the temporal correlation functions at two points in the IC and MS domains. The frequency spectra of the turbulent energy at these two points also demonstrate good agreement, as shown in Fig. 13, indicating that the turbulent energy for eddies of different temporal scales are correctly reproduced in IC domain as in the MS domain. This evidence clearly demonstrates that the large turbulent structures in the IC domain generated by the R^2^M technique have the correct spatial and temporal scales as in the naturally developed boundary layer in the MS domain, which also agrees with the available experimental data. **N.B.** The oscillations in the spectrum at *x*=−7.5*δ* (i.e. within the IC domain) at non-dimensional frequencies of 0.2, 0.4, 0.6 and 0.8 in Fig. 13 are an artefact of the rescaling procedure as noted in the Introduction. In this simulation, the flow in the IC domain is rescaled every 10 time steps; thus the rescale frequency is *f*_*R*_ = *f*_*s*_/10 (*f*_*s*_ is the inverse of one LES time step), which corresponds to a non-dimensionalised frequency 2*f*_*R*_/*f*_*s*_=0.2 on the x-axis of Fig. 13. According to standard signal processing theory, rescaling effects will necessarily appear at multiples of *f*_*R*_ in the frequency spectrum. However, it can be observed that the numerical rescaling energy is several orders of magnitude smaller than the true turbulent energetic motions and thus in the present method and for the problem examined the rescaling procedure does not pollute the physical turbulent structures. The energy spectral peak associated with the recycling frequency (a low frequency) reported in [23] is not observed here.

**Fig. 14:**
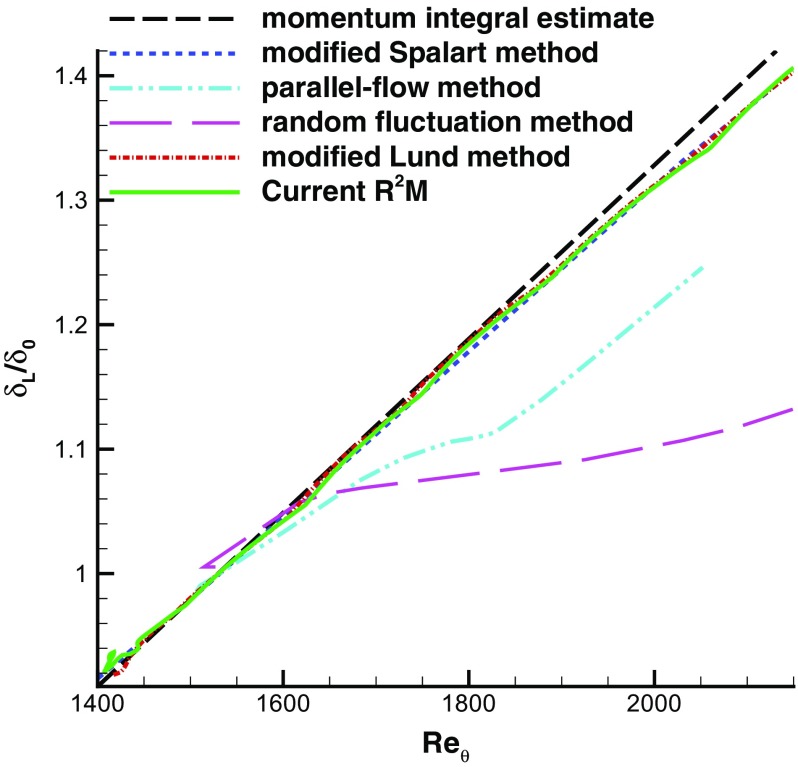
Evolution of the boundary layer thickness. Data of modified Spalart method, parallel-flow method, and random fluctuation method are from Lund et al. [19]

This method has generated an LES inlet condition which should require no (or very small) adjustment region for the flow in the MS domain. Figure 14 supports this conclusion by showing the evolution of the predicted boundary layer thickness with inflow conditions prescribed by different methods. In order to make comparison between the method proposed here with the methods investigated in Lund et al. [19], the simulated boundary layer thickness is normalised by *δ*_0_, the boundary layer thickness corresponding to *R* *e*_*𝜃*_=1530. The current R^2^M approach generally behaves as well as Lund et al.’s modified Spalart method, except that the boundary layer thickness with the current approach does not grow exactly linearly, but with occasional slight departure. blackOne simulation using Lund et al.’s method with modifications for easy implementation (referred to as modified Lund’s method and described in Appendix AA) has been run, and the predicted boundary layer thickness growth also shows trivial non-linearity in comparison with Lund et al. [19]. This implies that the occasional deviation of boundary layer thickness from Lund et al.’s result [19] may arise from the applied SGS model and simulation settings, and a dynamic SGS model [9] may improve the the performance of R^2^M. Although in the IC domain the target mean velocity and rms profiles corresponding to *R* *e*_*𝜃*_=1410 from [27] were used in R^2^M, at the MS inlet plane *x*/*δ*=0 the simulated *R* *e*_*𝜃*_ is 1435 due to the effect of the downstream flow. Near the MS inlet plane, there is a small adjustment region. This may be attributed to the standard Smagorinsky model which behaves poorly in the viscous wall region. Though the mean velocity in the IC domain achieves the target value because of the rescaling procedure, the mean velocity in the MS domain quickly develops to that consistent with the standard Smagorinsky model. Because the standard Smagorinsky model erroneously predicts larger wall friction, a dynamic SGS model would perhaps reduce these small adjustment effects.

### LES of a non-equilibrium turbulent boundary layer

**Fig. 15:**
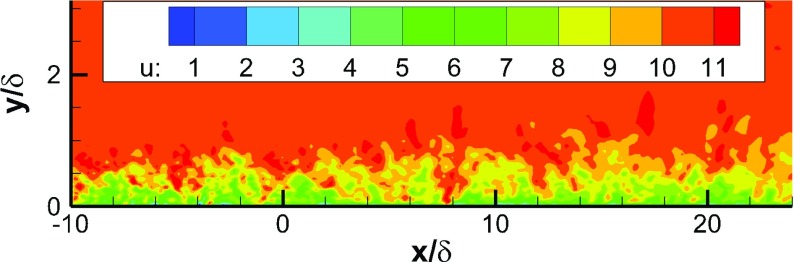
Contours of the streamwise velocity *U* in an *x*−*y* plane of a turbulent boundary layer simulation with recycling and rescaling method as inflow conditions for the region downstream of *x*/*δ*=0

**Fig. 16:**
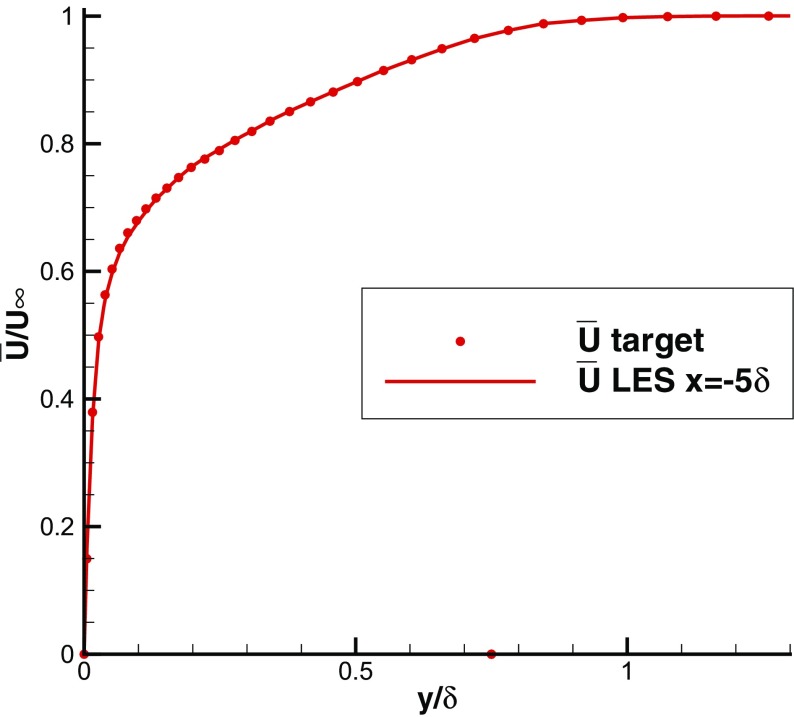
Mean velocity profile in the IC domain (*x*=−5*δ*) predicted in LES with unrealistic inflow rms intensity

**Fig. 17:**
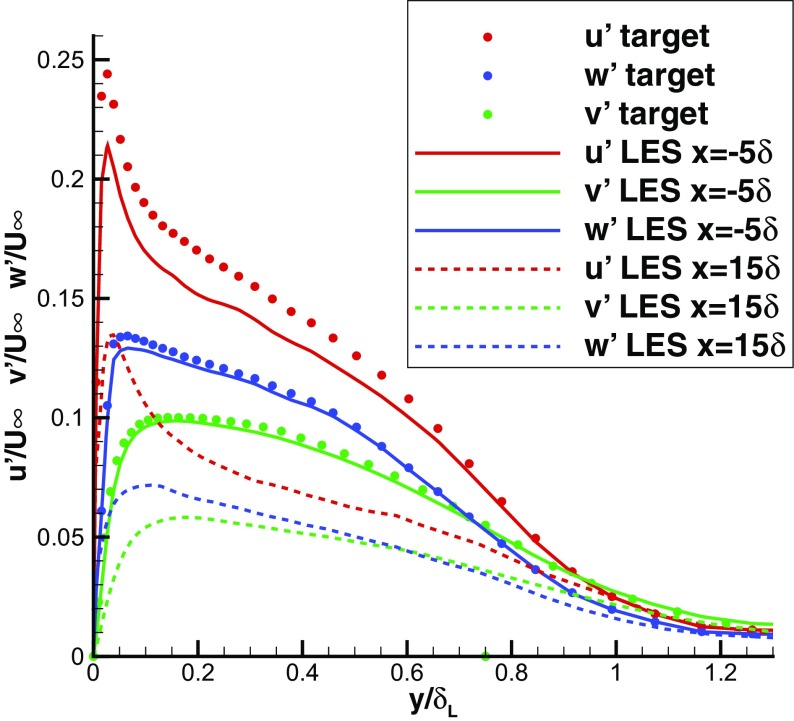
rms intensity in the IC domain (*x*=−5*δ*) and MS domain (*x*=15*δ*) predicted in LES with unrealistic inflow rms intensity

Lund et al.’s method [19] and many others are developed for a naturally developing turbulent boundary layer where the similarity law can be made use of to rescale the data at the recycling plane and to recycle them back to the inlet plane. However, there are situations in industrial applications where the flow at the MS inlet boundary has not reached equilibrium state and the similarity law is not satisfied. The capability of R^2^M to produce a turbulent inflow that is not in the equilibrium state is explored in this section. To investigate this, the target rms intensities were set to be twice those used in Section 3.1 while the mean velocity profile remained the same. Statistically stationary results could still be obtained, and Fig. 15 shows the predicted instantaneous contours of the streamwise velocity *U* in an *x*−*y* plane. Coherent turbulent structures are numerically produced in the IC domain. By comparing Fig. 15 with Fig. 5, it may be observed that within the IC domain more regions with *U*>11*m*/*s* were created with the altered target data input and these regions penetrated deeper into the boundary layer; this is clearly consistent with the higher turbulence intensity specified. Figures 16 and 17 demonstrate that both mean velocity and rms normal stresses collapsed onto their target profiles, although the fit of the streamwise rms was slightly worse than that achieved when stress levels from a developing boundary layer were specified (see Fig. 7). Since the specified high turbulence intensity in the IC domain cannot be sustained in a naturally developing turbulent boundary layer, the turbulence intensities decrease gradually towards their natural level in the MS domain as shown in Fig. 17. Therefore, R^2^M can produce non-equilibrium turbulent inflows with turbulence intensity significantly deviating from that in a naturally developing flow.

### LES of a turbulent mixing layer

The turbulent mixing layer studied experimentally by Tageldin and Cetegen [31] is chosen as the next test case. A splitter plate is inserted in the middle of a wide rectangular channel. On the lower side is a high speed flow with a mean velocity of 7.1*m*/*s* whilst on the upper side is a low speed flow with a mean velocity of 2*m*/*s*. After the trailing edge of the splitter a mixing layer develops and is investigated within the test section 0*m* *m*≤*x*≤200*m* *m*. Since the turbulent boundary layers on either side of the splitter significantly affect the initial development of the mixing layer, proper inflow conditions must be prescribed at the inlet-plane *x*=0 to obtain a correct prediction of the early shape of the mixing layer,

It is reported in [31] that the boundary layer of the fast stream has a momentum Reynolds number (*R* *e*_*𝜃*_) of 244.5 at the end of the splitter, but there are no experimental data for mean velocity or rms profiles for the wall boundary layers reported for this test case in the paper. Since the mean velocity and rms normalised by free stream velocity should have similar profiles at similar *R* *e*_*𝜃*_, the non-dimensional profiles corresponding to *R* *e*_*𝜃*_=300 from DNS of Spalart [27] are used to create the target data.

**Fig. 18:**
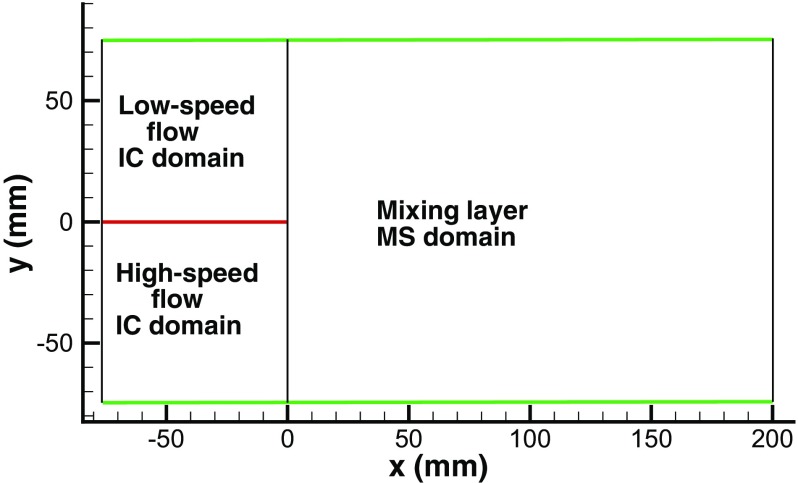
Simulation domain for a mixing layer with inflows generated by R^2^M. Green lines represent the wall, and the red line represents the splitter

Figure 18 shows the simulation domain for a mixing layer with inflows generated by R^2^M. Two IC domains are created respectively for the high-speed and low-speed flows on either side of the splitter to generate the inflow conditions at the MS inlet-plane *x*=0. The streamwise and spanwise sizes of the IC domains are:

12 $$ L_{x}/\delta_{BL}=4\pi; \qquad L_{z}/\delta_{BL}=7.5 $$

where *δ*_*BL*_ is the 99 % velocity thickness of the wall boundary layer developed on the splitter plate by the high-speed flow.

**Fig. 19:**
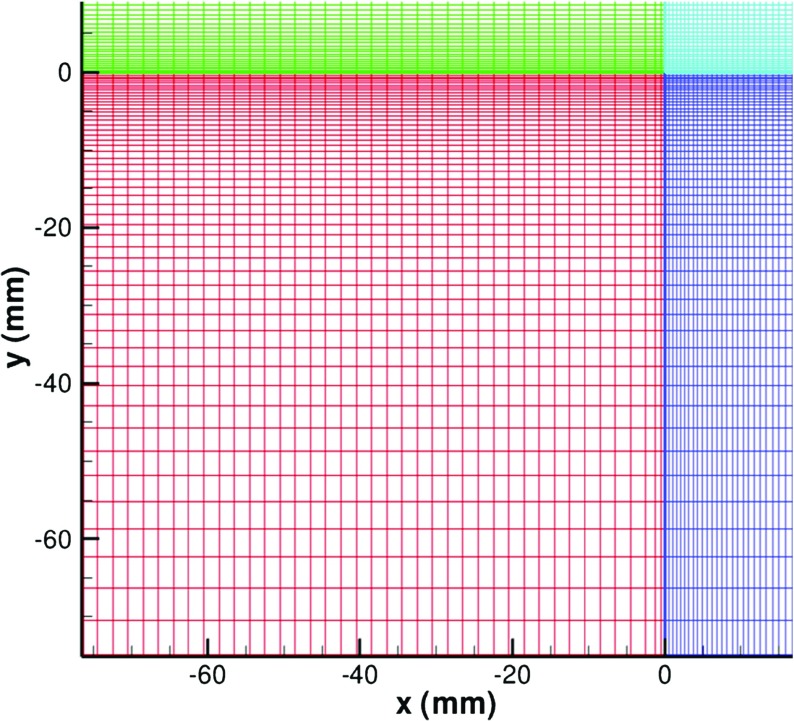
Mesh in the IC domains

Periodic conditions are used in the spanwise direction. In this test case, the velocity field within the IC domains is rescaled every 10 time steps. Figure 19 shows the mesh in the IC domains. The mesh is uniform in the streamwise direction except that the few meshlines near the MS inlet-plane become finer to match the mesh in the MS domain where a fine x-mesh is required to resolve the initial development of two wall boundary layer regions into a free shear layer. The uniform mesh upstream in the IC domains is required by the recycling and rescaling technique. The velocity from the plane *x*=−*δ*_*BL*_ is recycled to provide inflow conditions for the IC domains.

To investigate the effects of inflow conditions on the development of the mixing layer, an extra simulation with inflows generated by a simple white noise method has been performed. In this simulation, the inflows are specified directly at the inlet of the IC domains, and the IC domains are retained in an attempt to recover some turbulent boundary layer growth on the splitter plane.

**Fig. 20:**
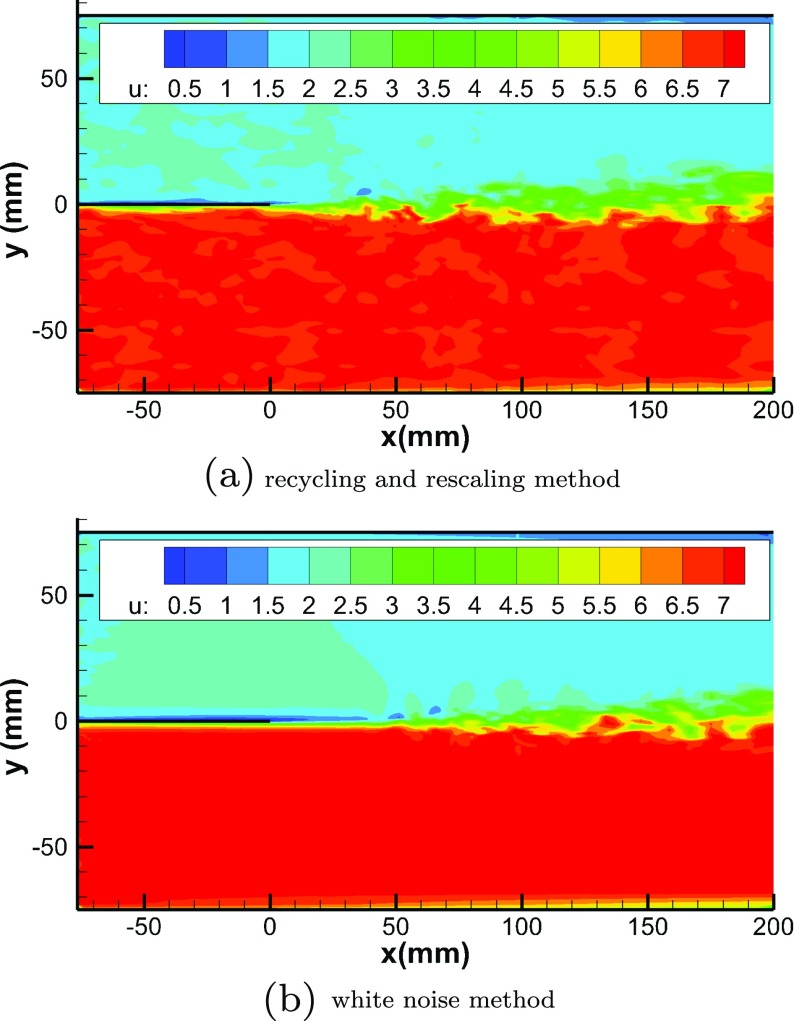
Contours of streamwise velocity *U* in the *x*−*y* plane of mixing layer simulation with two different inflow conditions generation techniques

Figure 20 shows contours of the streamwise velocity *U* in an *x*-*y* plane predicted using the two inflow generation methods. Using the proposed R^2^M, realistic turbulent structures are generated in the IC domains and convected into the MS domain, and as a consequence the mixing layer begins to develop immediately after the splitter trailing edge. However, with the white noise method, the perturbation decays immediately after the IC inlet plane, no realistic turbulence is generated at the splitter trailing edge, and the mixing layer only begins to develop from ∼50*m* *m* downstream of the splitter trailing edge. blackNote that close examination of the contours in the freestream region on the high speed side in Fig. 20(a) reveals the presence of periodically appearing large structures. These are mainly due to large eddies created by the low intensity freestream turbulence specified (2 % of the freestream velocity) which dissipate only slowly and are convected downstream.

Assuming that the free stream velocities of the high speed and low speed flows are *U*_*max*_ and *U*_*min*_ respectively, the velocity difference is △*U* = *U*_*max*_−*U*_*min*_. *y*_0.5_ is the locus of the mixing layer centreline where *U* = *U*_*min*_+△*U*/2. The velocity thickness *δ* of the mixing layer is defined as the distance between the locus where *U* = *U*_*min*_+10 *%*△*U* and *U* = *U*_*min*_+90 *%*△*U*.

**Fig. 21:**
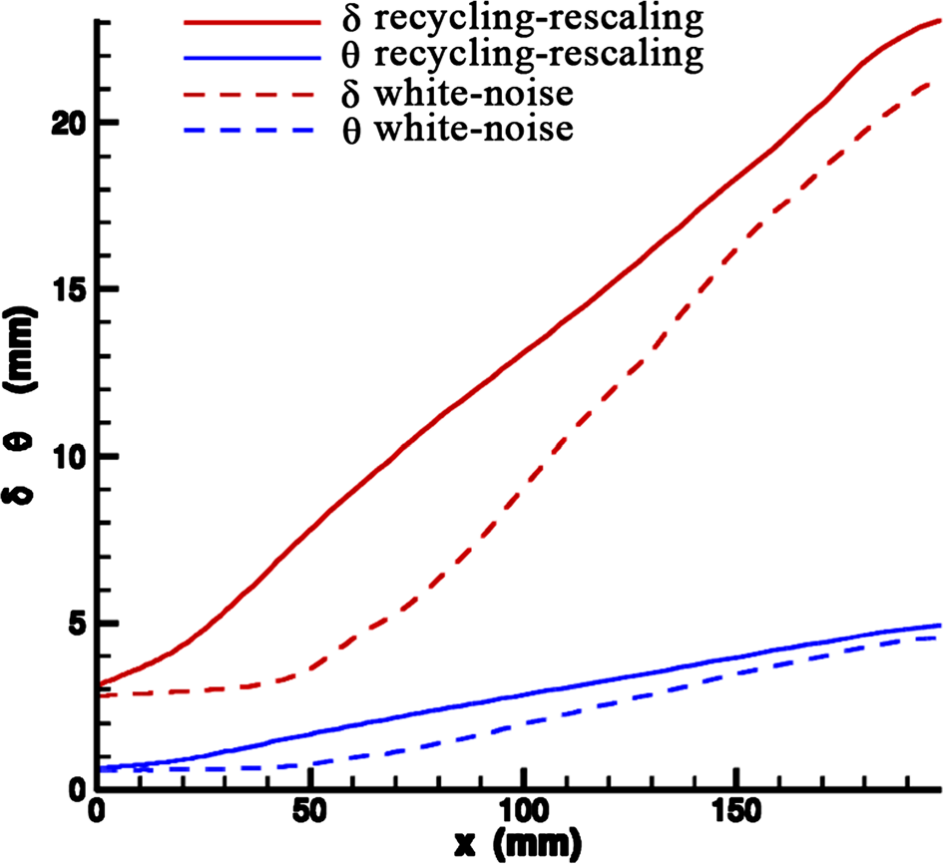
Evolution of the velocity thickness *δ* and the momentum thickness *𝜃* of the mixing layer in the streamwise direction

Figure 21 shows the evolution of this velocity thickness *δ* and the momentum thickness *𝜃* of the mixing layer in the streamwise direction. When using a white noise method, the velocity and momentum thickness begin to grow linearly only from 50*m* *m* downstream of the trailing edge of the splitter; with R^2^M, the correct growth rates of velocity and momentum thickness are observed right after the trailing edge of the splitter. blackThe white noise method does not clearly display the correct growth rate until perhaps 130*m* *m* downstream, showing how long adjustment lengths can be caused by poor inlet turbulence specification.

**Fig. 22:**
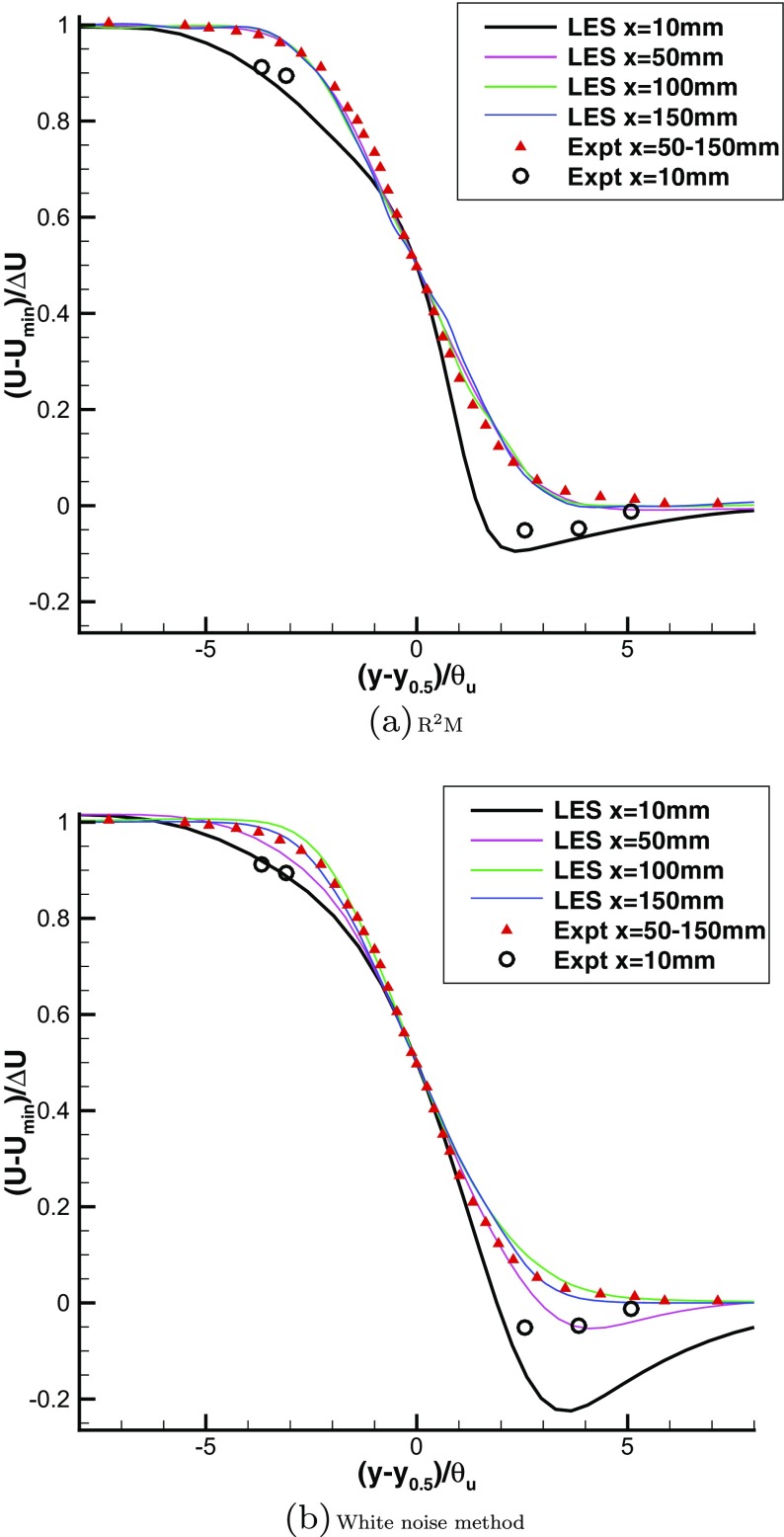
Predicted mean velocity profiles with two different inflow generation methods

Figure 22 shows measured and predicted streamwise mean velocity distributions in similarity coordinates. No experimental measurements are available for the gaseous mixing layer at the simulated flow condition, but experimental data at the closely similar flow condition (a shear parameter (*U*_*high*_−*U*_*low*_)/(*U*_*high*_ + *U*_*low*_) of 0.615, only 9 % higher than in the case simulated) are given in [31] and are thus used here. When using the proposed recycling and rescaling method to generate the inflow condition, the mean velocity profiles at locations x=50, 100, 150mm collapse well onto a single distribution in universal mixing layer coordinates, agreeing well with the experimental values, indicating that the mean velocity distribution has quickly reached self-similarity. The R^2^M-predicted mean velocity profile at location x=10mm displays significant departure from similarity and this also agrees well with experimental data. However, with the white noise method, the mean velocity distributions only showed similarity after 100mm downstream, and the mean velocity profiles at locations x=10, 50mm deviate considerably from the experiment. blackIt is also worthwhile commenting that the agreement of the current R^2^M prediction with measurements is considerably better than the results of Jones et al. [12] for the same test case. The Jones et al. [12] data display similarity already at the 10mm station, probably because the LES inlet condition treatment adopted (using a digital filter synthetic method) is described as fully developed, although it is unclear what this means for a spatially developing boundary layer as present on the splitter plate in this experiment.

**Fig. 23:**
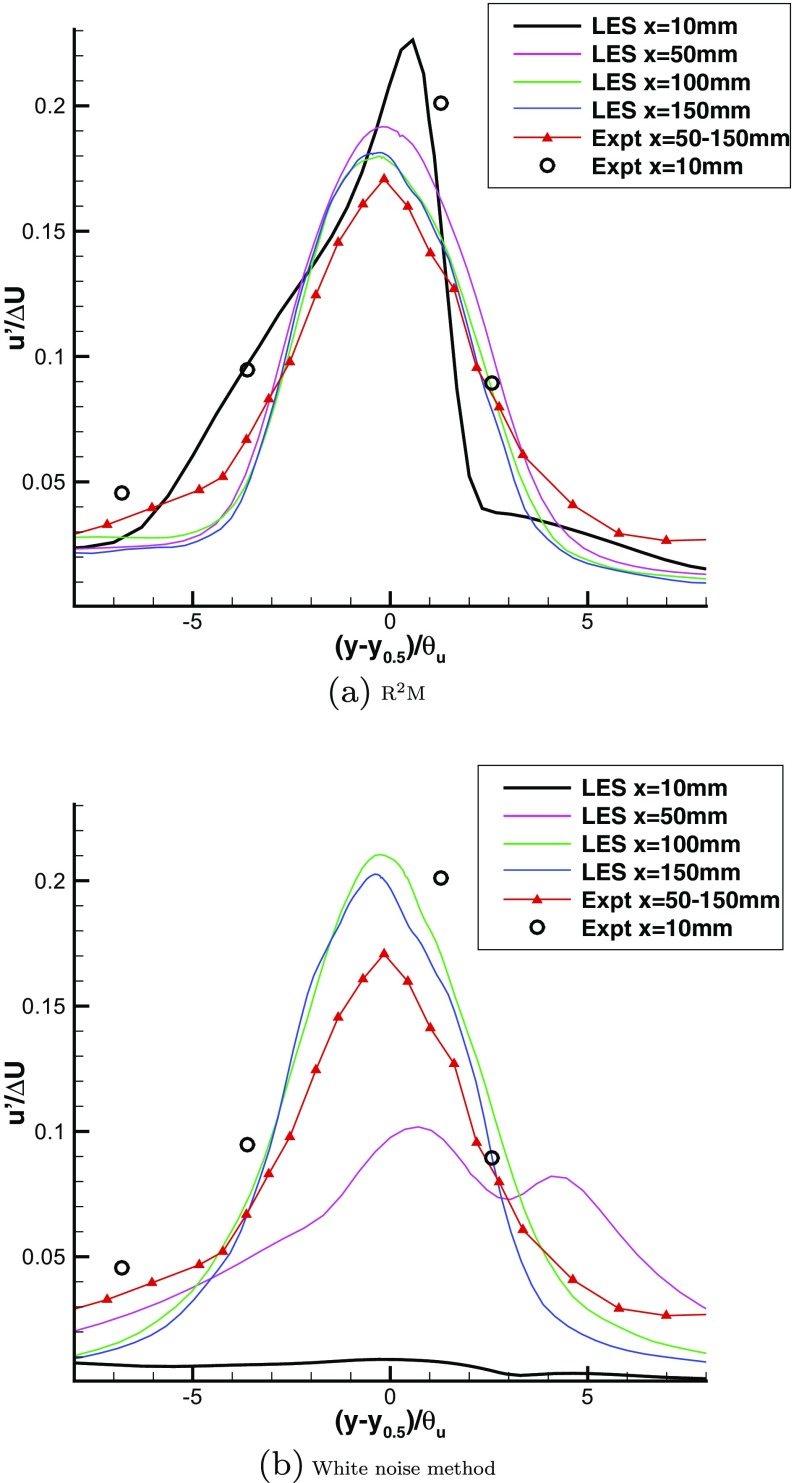
Predicted *u*−*r* *m* *s* profiles with two different inflow generation methods

Figure 23 shows measured and predicted distributions for the turbulence intensity *u*-*rms*. When using the present method, the rms profiles at locations x=50, 100, 150mm generally collapse onto a single distribution in universal mixing layer coordinates, agreeing also quantitatively with the experimental values. The rms profile at location x=10mm is also correctly predicted by LES, in comparison with the experiment. In contrast, the performance of the white noise method is very poor; there is almost no turbulence at location x=10mm. Though turbulence begins developing at x=50mm, the rms is still much lower than the experimental value, and the profile disagrees qualitatively with that from the experiment. The rms profiles at locations x=100, 150mm generally collapsed together, but showing much higher peak values than the experimental data.

Comparing the two simulations with inflow generated either by R^2^M or the white noise method, the additional cost per time step with R^2^M is less than 0.5 *%*, since the time required by the recycling and rescaling algorithm is negligible in comparison with that spent on solving the pressure Poisson equation. Realistic turbulent inflow can be reproduced in two IC domain flow-through times by R^2^M. This is also significantly shorter than the tens of MS domain flow-through times required to obtain the statistics of the turbulent mixing layer. Therefore, the total additional cost arising from R^2^M is no more than 10 %.

### Generation of spanwise inhomogeneous inflow

In this section, artificial mean velocity and rms target profiles were prescribed to demonstrate the capability of R^2^M to handle spanwise inhomogeneous inflow, as would apply for a 3D boundary layer for example. The mean velocity and rms target profiles in Section 3.3 were hereby multiplied by a factor $1+0.1127\cos (z\pi /L_{z})$ to generate spanwise inhomogeneity:

13 $$ \bar{U}_{i,\thinspace target}(y,z)=\bar{U}_{i,\thinspace target}(y)(1+0.1127\cos^{2}(z\pi/L_{z})) $$

14 $$ {u}_{i,\thinspace target}^{\prime}(y,z)={u}_{i,\thinspace target}^{\prime}(y)(1+0.1127\cos^{2}(z\pi/L_{z})) $$

where $\bar {U}_{i,\thinspace target}(y)$ and ${u}_{i,\thinspace target}^{\prime }(y)$ are the target profiles used for the turbulent boundary layer on the high speed flow side of the mixing layer test case in Section 3.3; *L*_*z*_ is the spanwise size of the IC domain, and has a value of 42*m* *m*. Since the multiplying factor is periodic, periodic boundary conditions can still be applied in the spanwise direction. For this test case, only the flow in the IC domain was simulated for demonstration, and periodic boundary conditions were also used in the streamwise direction.

**Fig. 24:**
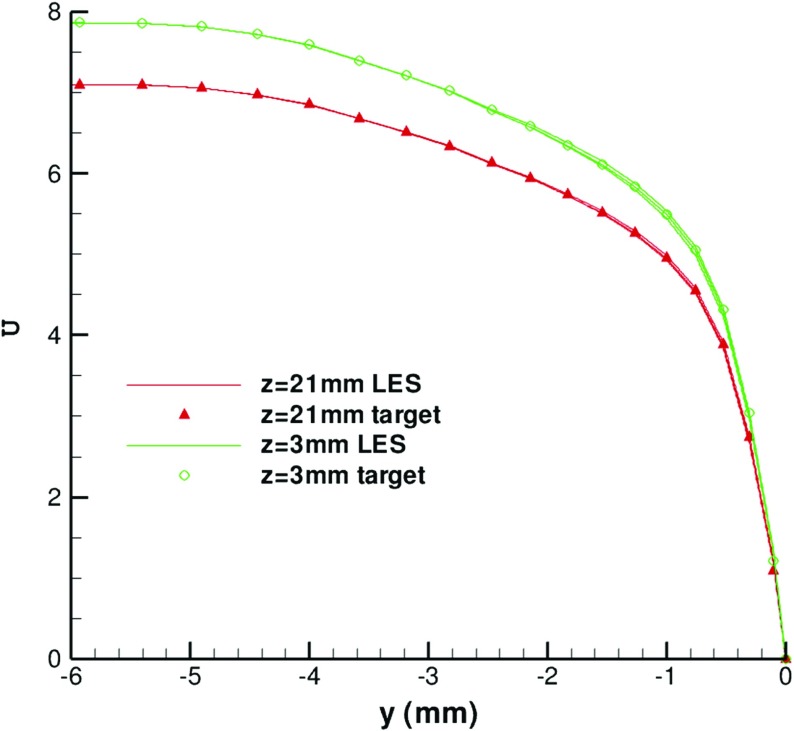
Mean velocity profiles at different spanwise locations: *z*=3*m* *m*, *z*=21*m* *m*

Figure 24 shows the generated mean velocity profiles at two spanwise locations: *z*=3*m* *m* and *z*=21*m* *m* and at three streamwise locations: *x*=−70*m* *m*, *x*=−40*m* *m*, and *x*=−10*m* *m*. At each spanwise location, the mean velocity profiles at the three different streamwise locations collapse onto the target values, indicating that the flow is homogeneous in the streamwise direction.

**Fig. 25:**
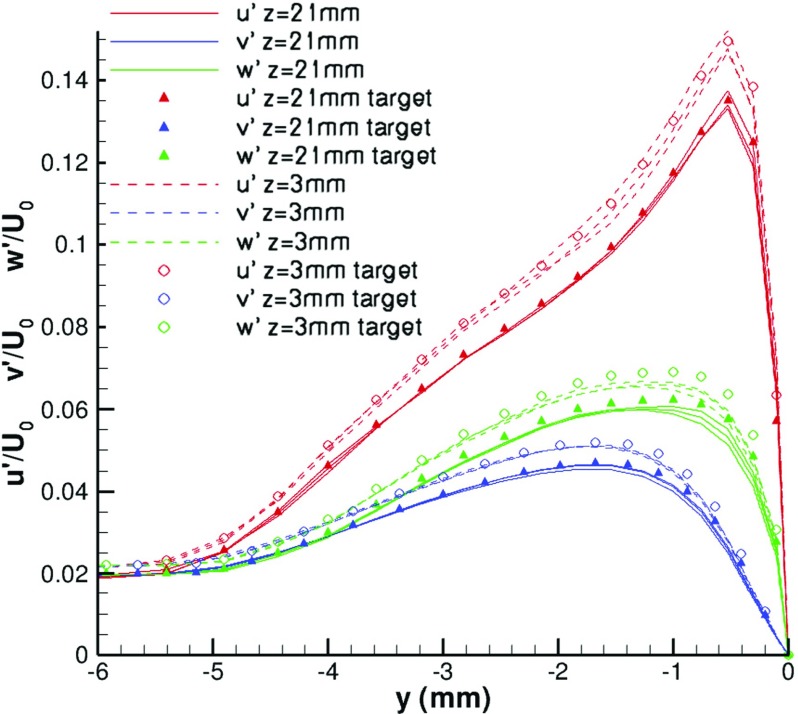
Rms profiles at different spanwise locations: *z*=3*m* *m*, *z*=21*m* *m*

Figure 25 shows the rms profiles at two spanwise locations: *z*=3*m* *m* and *z*=21*m* *m* and at three streamwise locations: *x*=−70*m* *m*, *x*=−40*m* *m*, and *x*=−10*m* *m*. At each spanwise location, the rms profiles of the three different streamwise locations generated by R^2^M agree well with their target values.

### LES of droplet-laden turbulent mixing layer

blackAs a final illustration of the performance of the current R ^2^M technique, the experiments of Tageldin and Cetegen [31] are considered. This experiment comprised the same gaseous mixing layer as studied in Section 3.3, but now the high-speed flow of the mixing layer was seeded with liquid droplets. The liquid volume flux and Sauter mean diameter (SMD) were measured at the plane *x*=0 (i.e. the splitter plate trailing edge) in the experiments, and thus the droplets were also released at this plane in the LES. In order to reduce the computational cost, the droplets were tracked only in the region 0≤*x*≤170*m* *m*,−40*m* *m*≤*y*≤40*m* *m*. As to inlet conditions for droplets, four segments with specified liquid volume flux and SMD as in Table 1 were used to account for the effect of the wall boundary layer on the droplet distribution. Since relaxation of droplet motion to the local gas velocity was achieved at the splitter plate end as mentioned in [31], the initial velocity of droplets is set to the local gas velocity. In order to investigate the influence of the treatment of turbulence at the inflow and the significance of SGS droplet dispersion, three simulations were run: (i) an LES using white noise for inflow fluctuations and the SGS droplet dispersion model (LES-WNM), (ii) an LES using R^2^M and the SGS droplet dispersion model (LES- R^2^M), and (iii) an LES using R^2^M but no SGS droplet dispersion model (LES- R^2^M-N).

**Table 1:** Four segments for droplet inlet condition

Segment number	Region (*mm*)	Liquid volume flux (*c* *c*/(*c* *m* ^2^⋅*s*)×10^−3^)	SMD
1	−40≤*y*≤−4.5	2.35	31.66
2	−4.5≤*y*≤−2.3	1.92	26.76
3	−2.3≤*y*≤−0.8	1.4	21.87
4	−0.8≤*y*≤0	0.6	18.80

**Fig. 26:**
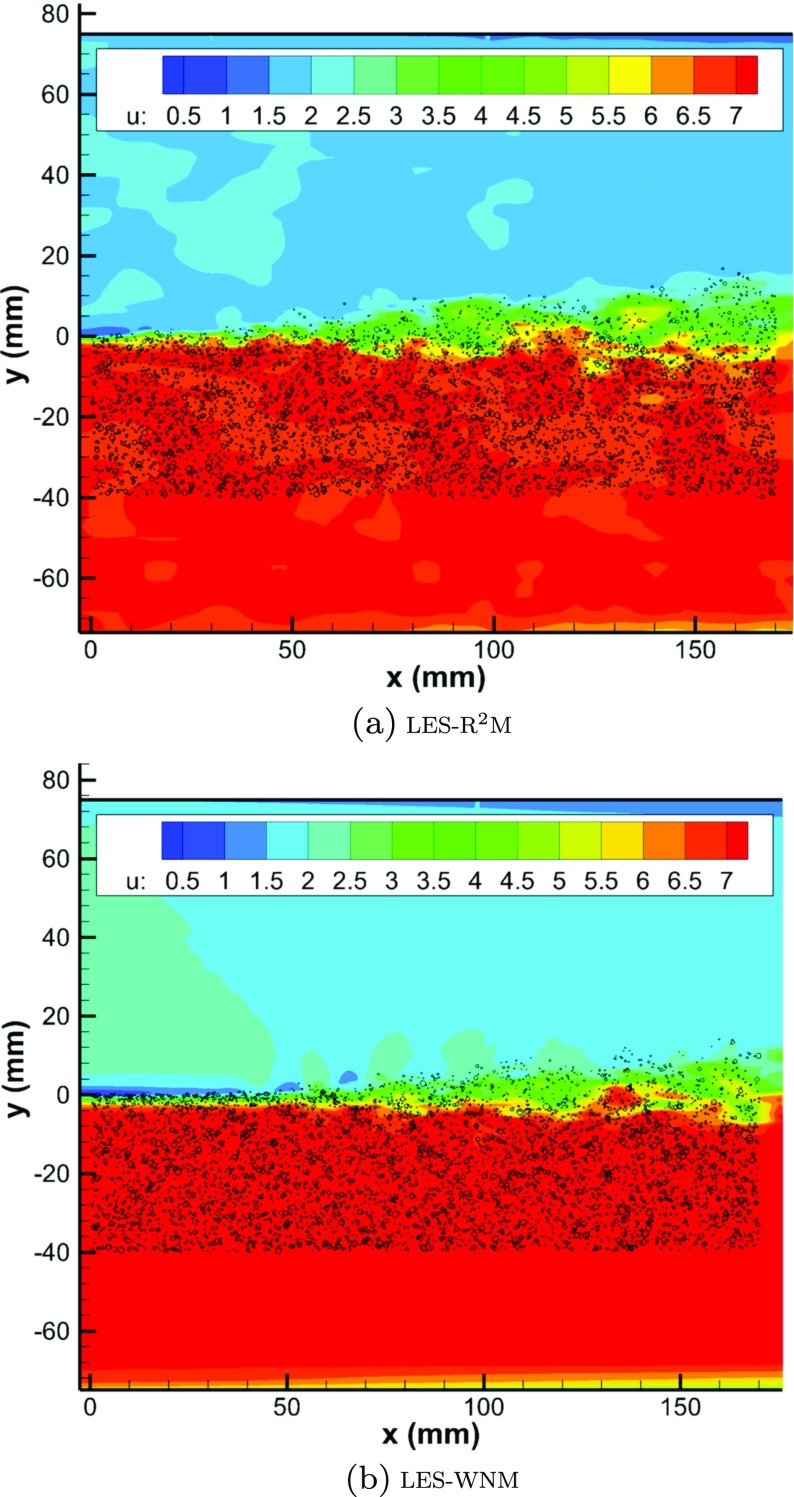
Instantaneous image of droplets distribution. (Cirlce size is proportional to droplet diameter)

**Fig. 27:**
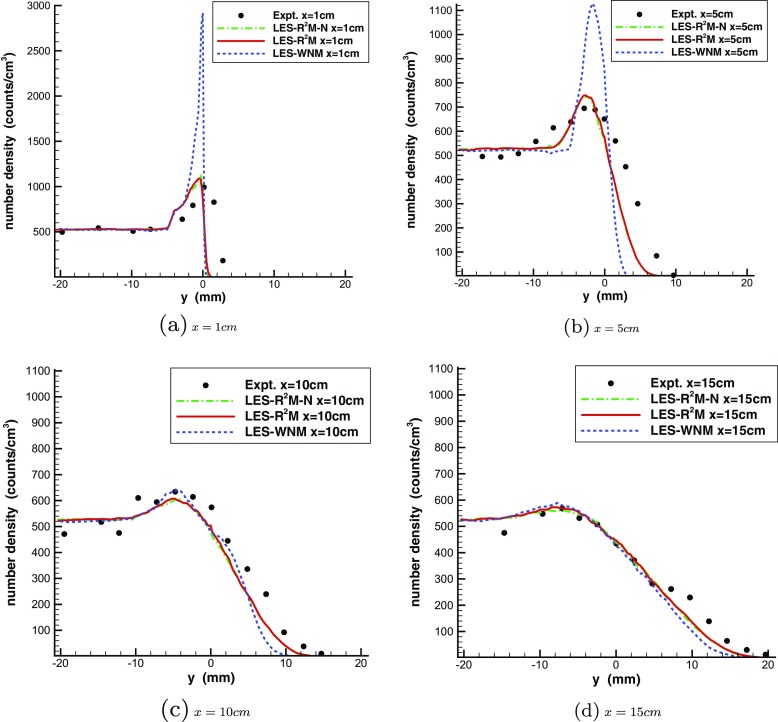
Droplet number density distribution in the turbulent mixing layer

The instantaneous droplet locations are shown in Fig. 26, demonstrating that the droplets dispersion is determined by the large turbulent structures of the mixing layer. When the white noise method is used, the droplets are observed to cluster in the laminar mixing layer in the region 0<*x*<5*c* *m*. Figure 27 gives a quantitative comparison of droplet number density distribution between LES predictions and experimental results. The peak of the droplet number density predicted by LES-WNM at *x*=1*c* *m* and *x*=5*c* *m* is much higher than the experimental measurements due to the preferential concentration of droplets in the laminar mixing layer. Since realistic turbulent inflows are generated by R^2^M, allowing a turbulent mixing layer to be reproduced right after the splitter trailing edge in LES- R^2^M, the simulated droplets are now dispersed more widely due to the turbulent vortices in the region 0<*x*<5*c* *m*, resulting in an improved droplet number density distribution with a correct peak value at *x*=1*c* *m* and *x*=5*c* *m*. The discrepancy between LES- R^2^M and experimental data at location *x*=1*c* *m* is due to the fact that the splitter has a thickness of 0.6mm at the trailing edge in the experiment while in the simulation the splitter is treated as having zero thickness. In the further downstream region *x*>5*c* *m*, the turbulent mixing layer begins to develop in LES-WNM and the droplet dispersion in the turbulent mixing layer can be observed, producing approximately the same peak value of droplet number density as the experiment and LES- R^2^M at *x*=10*c* *m* and *x*=15*c* *m*. Figure 27 demonstrates that the droplets predicted by LES- R^2^M penetrate further into the low-speed side than LES-WNM at all four locations, agreeing better with the experimental measurements. Note also that the difference between the droplet number density distributions predicted by LES- R^2^M and LES- R^2^M-N is very small. This implies that SGS droplet dispersion is negligible. This result is in contrast to the findings of Jones et al. [12] who observed that in their LES calculations of this test problem the SGS dispersion model was essential to obtain close agreement with the droplet spreading rate (although they also reported the strange result that an increase of the coefficient *C*_0_ in the SGS dispersion model by a factor of 4 had little effect). The probable explanation for this is that the LES mesh used in the present simulations is considerably finer in the mixing layer (filter width Δ=0.42*m* *m*)than that used in [12] where Δ=0.9*m* *m*, resulting in a low SGS kinetic energy in the gas mixing layer and thus reducing the importance of SGS dispersion for droplets.

**Fig. 28:**
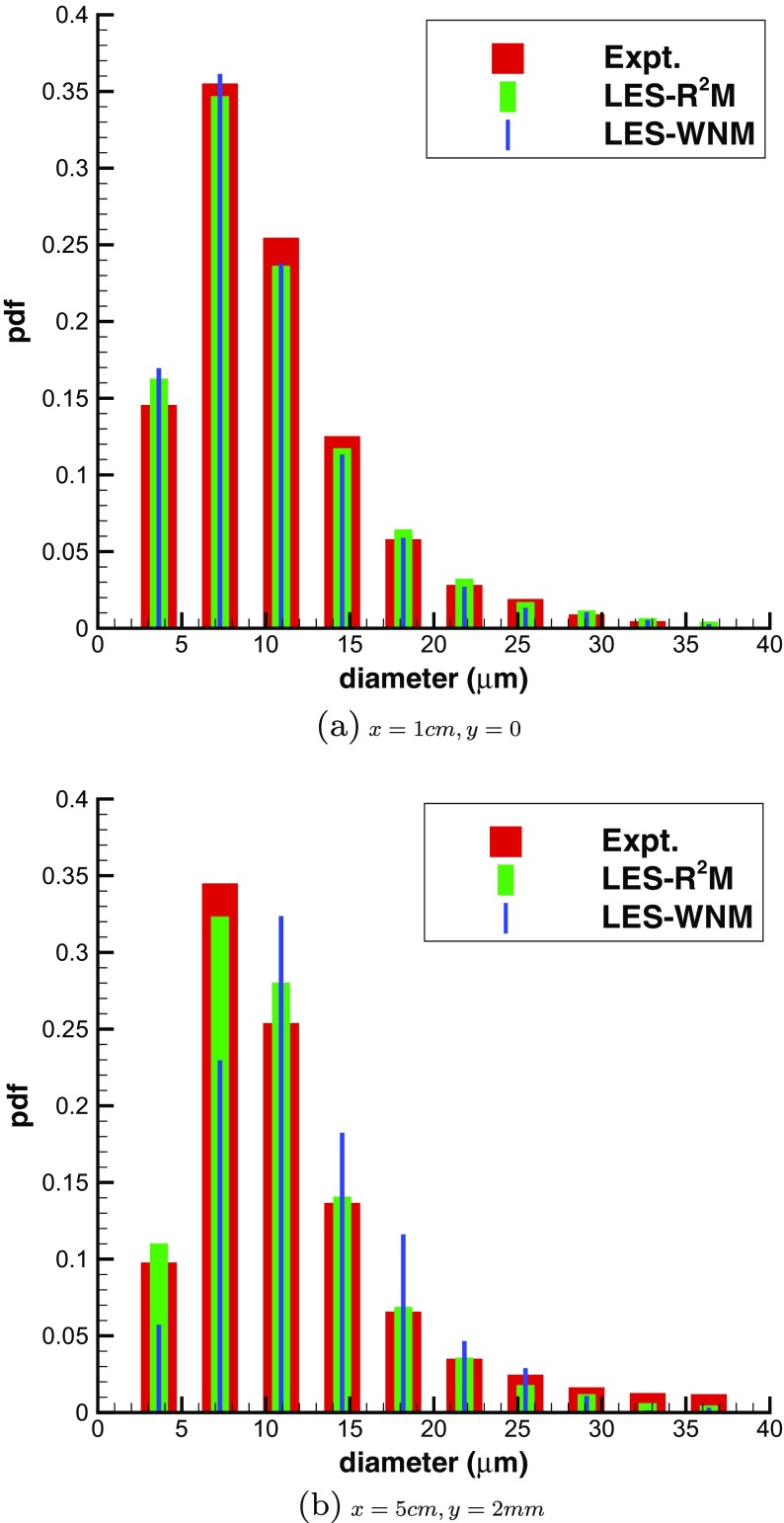
Droplet size distribution in the mixing layer

Finally, Fig. 28 shows the size distribution of droplets entrained into the turbulent mixing layer at two downstream stations in the shear layer near-field. LES-WNM and LES- R^2^M show a similar level of accuracy in predicting droplet size distribution at the first station (*x*=1*c* *m*,*y*=0) in comparison with the experiment, because this is close to the splitter plate trailing edge and the distributions are essentially unchanged from the inlet condition. Further downstream and further away from the splitter plate location (at *x*=5*c* *m*,*y*=2*m* *m*), the droplet size distribution predicted by LES- R^2^M agrees significantly better with experimental measurements than the white noise simulation, emphasizing the benefit of an accurate turbulence inlet condition for the dispersion of a dispersed second phase as well as for the carrier phase velocity field.

## Conclusion

The work presented here has demonstrated a modified R^2^M approach for LES inflow condition generation. With only mean and rms values over the inlet plane supplied as input, this method enables realistic and consistent turbulent structures to be generated. It has been validated by applying it to a spanwise homogeneous turbulent boundary layer and a turbulent mixing layer. Furthermore, it was shown how the method can be applied to more complex inflow profiles as shown by successful generation of spanwise inhomogeneous inflow. blackWith the realistic turbulent inflow produced by the R^2^M approach it was also applied to a two-phase flow, namely droplet dispersion across a turbulent mixing layer; the results showed how the near-field dispersion was significantly better predicted using LES driven by the current inflow generation method. The primary disadvantage of the approach is the cost involved in carrying out a separate LES in the inlet condition domain. However, it is argued that the benefits of the method are sufficient to make this increased cost worthwhile. These benefits are: (i) it is not restricted to naturally developing turbulent boundary layer flow where similarity laws are available as in Lund et al.’s method [19], and it can produce non-equilibrium turbulent inflow; (ii) the method can cope with complex inflow conditions involving spatial variations in both directions across the inlet plane, (iii) only mean velocity and turbulence normal stress variations in the inlet plane are needed, (iv) the method can generate the unspecified 1-point and 2-point statistical quantities that are consistent with the input data (e.g. Reynolds shear stresses, 2-point spatial and temporal correlations, integral length scales), (v) only a short start-up transient and very short adjustment length were observed in the test case flows considered, with no evidence of contamination by the numerical aspects of the procedure, e.g. errors associated with the recycling frequency.

Since the recycling/rescaling procedure and the choice of the SGS model used in the LES solution procedure are independent choices, there is no obvious reason why the approach should not work with dynamic SGS models. Hopefully, better results can be expected with the more advanced dynamic SGS models, but this remains to be tested.

## Appendix A: Modified Lund’s method

Lund et al.’s method [19] is implemented in our code but with several modifications following the application of Lund et al.’s method in supersonic flows in [8, 34, 36, 41]. The inflow generation simulation and the main simulation are merged here. The same mesh as that in Section 3.1 is used. The inlet and recycling planes are respectively *x*=−10*δ* and *x*=0. For easy implementation of the method, a few simplifications are made as follows (let *u* denote the instantaneous streamwise velocity, and $u=U+u^{\prime }$ where *U* and $u^{\prime }$ are respectively the mean and instantaneous fluctuation as in Lund et al. [19]):

1. It is argued in [11] that the boundary layer thickness *δ* is a troublesome quantity to calculate from the simulated mean velocity profile. In [34, 36, 41], *δ*_*r**e**c**y*_/*δ*_*i**n**l**t*_ (*δ*_*i**n**l**t*_ and *δ*_*r**e**c**y*_ are respectively the boundary thickness at the inlet and recycling plane) is computed from classical empirical correlations. For simplicity in the current simulation, *δ*_*r**e**c**y*_/*δ*_*i**n**l**t*_=1.2 is used by measuring the data in [19], and *γ* = *u*_*τ*,*i**n**l**t*_/*u*_*τ*,*r**e**c**y*_=(*δ*_*r**e**c**y*_/*δ*_*i**n**l**t*_)^1/8^ (*u*_*τ*,*i**n**l**t*_ and *u*_*τ*,*r**e**c**y*_ are respectively the friction velocity at the inlet and recycling plane) is used as in [19].
2. Following the application of the recycling/rescaling technique in [41], the mean streamwise velocity profile at the inlet is fixed rather than using the rescaled mean velocity from the recycling plane. The mean velocity of a boundary layer with *R* *e*_*𝜃*_=1410 from the DNS of Spalart [27] (*U*_*t**a**r**g**e**t*_) is used as in Section 3.1.
3. The streamwise velocity fluctuation is extracted at the recycling plane, and rescaled and reintroduced at the inlet plane as in [19]. In Edwards et al.’s work [8], the recycled fluctuations is multiplied by a Klebanoff-type intermittency function to prevent excessive turbulence energy accumulation in the outer part of the boundary layer. In the current simulation, the recycled streamwise fluctuations is multiplied by the Klebanoff-type intermittency function to prevent any numerical fluctuation from polluting the freestream velocity at the inlet plane which can cause spurious mass inflow and numerical instability.

With the above changes, the instantaneous streamwise velocity at the inlet is specified by:

15 $$ u_{inlt}=U_{target}+\left[\left( u^{\prime}\right)_{inlt}^{inner}\left( 1-W\left( \eta_{inlt}\right)\right)+\left( u^{\prime}\right)_{inlt}^{outer}W\left( \eta_{inlt}\right)\right]F_{Kleb} $$

16 $$ \left( u^{\prime}\right)_{inlt}^{inner}=\gamma \left( u^{\prime}\right)_{recy}\left( y^{+}_{inlt},z,t\right) \qquad y^{+}=\frac{u_{\tau} y}{\mu} $$

17 $$ \left( u^{\prime}\right)_{inlt}^{outer}=\gamma \left( u^{\prime}\right)_{recy}\left( \eta_{inlt},z,t\right) \qquad \eta=\frac{y}{\delta} $$

18 $$ W(\eta)=\frac{1}{2}\left\{1+\tanh\left[\frac{\alpha(\eta-b)}{(1-2b)\eta+b}\right]/\tanh(\alpha) \right\} \qquad \alpha=4 \quad b=0.2 $$

19 $$ F_{Kleb}=\left[1+(\eta/C_{Kleb})^{6}\right]^{-1} \qquad C_{Kleb}=1.1 $$

The procedure for specifying the instantaneous vertical and spanwise velocity at the inlet is the same as in [19].

## References

[1] Antonia RA, Luxton RE (1971). The response of a turbulent boundary layer to a step change in surface roughness. J. Fluid Mech..

[2] Araya G, Castillo L, Meneveau C, Jansen K (2011). A dynamic multi-scale approach for turbulent inflow boundary conditions in spatially developing flows. J. Fluid Mech..

[3] Arolla SK (2016). Inflow turbulence generation for eddy-resolving simulations of turbomachinery flows. ASME. J. Fluids Eng..

[4] Baba-Ahmadi MH, Tabor G (2009). Inlet conditions for les using mapping and feedback control. Comput. Fluids.

[5] Batten P (2004). Interfacing statistical turbulence closures with large-eddy simulation. AIAA J..

[6] Crowe, C.T., Sommerfeld, M., Tsuji, Y.: Multiphase flows with droplets and particles CRC press (1998)

[7] Dianat M, Yang Z, Jiang D, McGuirk JJ (2006). Large eddy simulation of scalar mixing in a coaxial confined jet. Flow Turbul. Combust..

[8] Edwards JR, Choi JI, Boles JA (2008). Large eddy/reynolds-averaged navier-stokes simulation of a mach 5 compression-corner interaction. AIAA J..

[9] Germano M, Piomelli U, Moin P, Cabot WH (1991). A dynamic subgrid-scale eddy viscosity model. Phys. Fluids.

[10] Jarrin N, Benhamadouche S, Laurence D, Prosser R (2006). A synthetic-eddy-method for generating inflow conditions for large-eddy simulations. Int. J. Heat Fluid Flow.

[11] Jewkes JW, Chung YM, Carpenter PW (2011). Modifications to a turbulent inflow generation method for boundary-layer flows. AIAA J..

[12] Jones WP, Lyra S, Marquis AJ (2010). Large eddy simulation of a droplet laden turbulent mixing layer. Int. J. Heat Fluid Flow.

[13] Keating A, Piomelli U, Balaras E, Kaltenbach HJ (2004). A priori and a posteriori tests of inflow conditions for large-eddy simulation. Phys. Fluids.

[14] Kempf A, Klein M, Janicka J (2005). Efficient generation of initial- and inflow-conditions for transient turbulent flows in arbitrary geometries. Flow Turbul. Combust..

[15] Klein M, Sadiki A, Janicka J (2003). A digital filter based generation of inflow data for spatially developing direct numerical or large eddy simulations. J. Comp. Phys..

[16] Le H, Moin P, Kim J (1997). Direct numerical simulation of turbulent flow over a backward-facing step. J. Fluid Mech..

[17] Lee S, Lele SK, Moin P (1992). Simulation of spatially evolving turbulence and the applicability of Taylor’s hypothesis in compressible flow. Physics of Fluids A: Fluid Dynamics.

[18] Liu K, Pletcher RH (2006). Inflow conditions for the large eddy simulation of turbulent boundary layers: a dynamic recycling procedure. J. Comp. Phys..

[19] Lund TS, Wu XH, Squires KD (1998). Generation of turbulent inflow data for spatially-developing boundary layer simulations. J. Comp. Phys..

[20] di Mare L, Klein M, Jones WP, Janicka J (2006). Synthetic turbulence inflow conditions for large-eddy simulation. Phys. Fluids.

[21] Mayor SD, Spalart PR, Tripoli GJ (2002). Application of a perturbation recycling method in the large-eddy simulation of a mesoscale convective internal boundary layer. J. Atmos. Sci..

[22] McMullan WA, Gao S, Coats CM (2009). The effect of inflow conditions on the transition to turbulence in LES of spatially developing mixing layers. Int. J. Heat Fluid Flow.

[23] Morgan B, Larsson J, Kawai S, Lele SK (2011). Improving low-frequency characteristics of recycling/rescaling inflow turbulence generation. AIAA J..

[24] Nikitin N (2007). Spatial periodicity of spatially evolving turbulent flow caused by inflow boundary condition. Phys. Fluids.

[25] Pierce, C.D.: Progress-variable approach for large-eddy simulation of turbulent combustion. Dissertation for PhD Stanford University (2001)

[26] Schiller L, Naumann A (1933). Über die grundlegenden berechnungen bei der schwerkraftaufbereitung. Z. Ver. Dtsch. Ing..

[27] Spalart PR (1988). Direct simulation of a turbulent boundary layer up to Re = 1410. J. Fluid Mech..

[28] Spalart PR, Strelets M, Travin A (2006). Direct numerical simulation of large-eddy-break-up devices in a boundary layer. Int. J. Heat Fluid Flow.

[29] Spille-Kohoff, A.: Generation of turbulent inflow data with a prescribed shear-stress profile. In: Third AFOSR International Conference on DNS/LES , in DNS/LES Progress and Challenges, pp. 319–326. C. Liu, L. Sakell, and T. Beutner (Greyden, Columbus, OH, 2001) (2001)

[30] Tabor GR, Baba-Ahmadi MH (2010). Inlet conditions for large eddy simulation: a review. Comput. Fluids.

[31] Tageldin MS, Cetegen BM (1997). Development of mixing and dispersion in an isothermal, droplet-laden, confined turbulent mixing layer. Combust. Sci. Technol..

[32] Tang G, Yang Z, McGuirk JJ (2004). Numerical methods for large-eddy simulation in general co-ordinates. Int. J. Numer. Fluids.

[33] Tyacke JC, Tucker PG (2015). Future use of large eddy simulation in aero-engines. J. Turbomach..

[34] Urbin G, Knight D (2001). Large-eddy simulation of a supersonic boundary layer using an unstructured grid. AIAA J..

[35] Veloudis I, Yang Z, McGuirk JJ, Page GJ, Spencer A (2007). Novel implementation /assessment of a digital filter approach for generation of LES inlet conditions. Flow Turbul. Combust..

[36] Wang H, Wang Z, Sun M, Qin N (2014). Large eddy simulation based studies of jet–cavity interactions in a supersonic flow. Acta Astronaut..

[37] Wu X (2017). Inflow turbulence generation methods. Ann. Rev. Fluid Mech..

[38] Xiao F, Dianat M, McGuirk JJ (2013). Large eddy simulation of liquid-jet primary breakup in air crossflow. AIAA J..

[39] Xiao F, Dianat M, McGuirk JJ (2014). Large eddy simulation of single droplet and liquid jet primary breakup using a coupled level set/volume of fluid method. Atomization Sprays.

[40] Xiao F, Dianat M, McGuirk JJ (2014). LES of turbulent liquid jet primary breakup in turbulent coaxial air flow. Int. J. Multiphase Flow.

[41] Xiao X, Edwards JR, Hassan H, Baurle R (2003). Inflow boundary conditions for hybrid large eddy/reynolds averaged navier-stokes simulations. AIAA J..

